# Recapitulating porcine cardiac development *in vitro*: from expanded potential stem cell to embryo culture models

**DOI:** 10.3389/fcell.2023.1111684

**Published:** 2023-05-15

**Authors:** Hilansi Rawat, Jessica Kornherr, Dorota Zawada, Sara Bakhshiyeva, Christian Kupatt, Karl-Ludwig Laugwitz, Andrea Bähr, Tatjana Dorn, Alessandra Moretti, Monika Nowak-Imialek

**Affiliations:** ^1^ First Department of Medicine, Cardiology, Klinikum Rechts der Isar, School of Medicine and Health, Technical University of Munich, Munich, Germany; ^2^ German Center for Cardiovascular Research (DZHK), Munich Heart Alliance, Munich, Germany; ^3^ Regenerative Medicine in Cardiovascular Diseases, First Department of Medicine, Klinikum Rechts der Isar, School of Medicine and Health, Technical University of Munich, Munich, Germany; ^4^ Department of Surgery, Yale University School of Medicine, New Haven, CT, United States

**Keywords:** pig, heart development, porcine expanded pluripotent stem cells, cardiac progenitor cells, epicardial cells, cardiomyocyte, cardiac differentiation

## Abstract

Domestic pigs (*Sus scrofa*) share many genetic, anatomical, and physiological traits with humans and therefore constitute an excellent preclinical animal model. Fundamental understanding of the cellular and molecular processes governing early porcine cardiogenesis is critical for developing advanced porcine models used for the study of heart diseases and new regenerative therapies. Here, we provide a detailed characterization of porcine cardiogenesis based on fetal porcine hearts at various developmental stages and cardiac cells derived from porcine expanded pluripotent stem cells (pEPSCs), i.e., stem cells having the potential to give rise to both embryonic and extraembryonic tissue. We notably demonstrate for the first time that pEPSCs can differentiate into cardiovascular progenitor cells (CPCs), functional cardiomyocytes (CMs), epicardial cells and epicardial-derived cells (EPDCs) *in vitro*. Furthermore, we present an enhanced system for whole-embryo culture which allows continuous *ex utero* development of porcine post-implantation embryos from the cardiac crescent stage (ED14) up to the cardiac looping (ED17) stage. These new techniques provide a versatile platform for studying porcine cardiac development and disease modeling.

## Introduction

Cardiovascular diseases including congenital heart defects remain the leading cause of mortality worldwide. Our current knowledge of cardiogenesis is largely based on studies performed in rodents, owing to their accessibility, rapid reproducibility, and relatively low cost. However, results from these models do not always translate to humans due to significant differences in cardiac development and physiology (Maselli et al., 2022). There is an urgent need to establish alternative model systems more closely related to humans in terms fetal development, organ size, anatomy, and physiology. Large animal models offer a clear advantage compared to rodents. Non-human primates have the closest phylogenetic relationship to humans, but these models are limited due to their high costs, prolonged breeding time, and high ethical concerns (Stirm et al., 2022). However, pigs represent a valuable alternative, since important physiological parameters such as heart rate, cardiac structure, and contractile function closely resemble those of an adult human (Hughes, 1986; Lelovas et al., 2014; Romagnuolo et al., 2019).

Although anatomical atlases of both human and mouse heart development have been published, only two recent studies have investigated embryonic cardiac development of pigs (Gabriel et al., 2021; Lauschke et al., 2021). However, *in vitro* models of porcine cardiogenesis are still limited due to the lack of *bona fide* pluripotent stem cells (PSCs) in this species. Recently, we established porcine expanded potential stem cells (pEPSCs) (Gao et al., 2019), which possess long-term self-renewal, allow precise genome editing, and have the ability to contribute to both embryonic and extraembryonic lineages *in vitro* and *in vivo*, thereby representing a major advance for differentiation studies and future cell therapy applications. However, the efficient generation of cardiac lineages from pEPSCs has not been yet investigated.

Here, we provide an anatomical and molecular characterization of *in vivo* porcine cardiogenesis at embryonic days ED13, ED14, ED15, ED17 and ED19. We also established an *ex utero* culture system allowing faithful monitoring of porcine embryonic cardiac development. Finally, we describe protocols for the directed differentiation of pEPSCs into CPCs, CMs, epicardial cells, smooth muscle cells (SMCs), endothelial cells (ECs) and cardiac fibroblasts (FBs). These platforms open new possibilities for the development of autologous or allogeneic cell-based cardiac regenerative therapies.

## Materials and methods

### Animals

German Landrace gilts (7–9 months) from an approved local farm facility were used as embryo donors. Animals were transported to the Technical University Munich, Klinikum rechts der Isar animal facility for sample isolation. Embryos were harvested in the accordance with §4 German Animal Welfare legislation. Pregnant sows were euthanized using pentobarbital (Euthadorm, CP-Pharma, Germany) according to the manufacturer’s specifications and the uterus was explanted immediately after cardiac arrest. All experiments were performed with permission from the local regulatory authority.

### pEPSCs culture

Porcine EPSCs were maintained on 0.1% gelatin-coated plates (Sigma-Aldrich^®^, G1890) with mitotically inactivated mouse SNL76/7 feeder cells in porcine EPSC medium (pEPSCM) as previously described by Gao et al. (2019). The N2B27 basal media was supplemented with 0.2 μM CHIR99021 (Tocris, 4423), 0.02 μM A-419259 trihydrochloride (Tocris, 3914), 2.5 μM XAV939 (Sigma-Aldrich^®^, X3004), 50 μg/mL ascorbic acid (Sigma-Aldrich^®^, 49752-100G), 10 ng/mL LIF (Millipore, LIF1050), 20 ng/mL Activin A (Stemcell Technologies, 78001.1). Cells were enzymatically passaged every 4 days using TrypLE™ (Thermo Fisher Scientific, 12605010). To promote cell survival, Rho-associated coiled kinase (ROCK) inhibitor Y-27632 (Tocris, 1254) was added at a concentration of 5 μM for 24 h after passaging. Two different pEPSCs lines (pEPSCs-T4 and pEPSCs-T6) at different passages were used for differentiation experiments.

### hESCs culture

Human ESC line used in these studies was approved by the Ethics Commission of the TUM Faculty of Medicine (# 447/17S). Authorization to use the hESC-TN was granted by the Central Ethics Committee for Stem Cell Research of the Robert Koch Institute to AM (AZ 3.04.02/0131). Generation of the hESC-TN line was described in Zawada et al. (2023). hESCs were maintained on Matrigel-coated plates (Corning, 354277) in Essential 8 medium (Thermo Fisher Scientific, A1517001) containing 0.5% penicillin/streptomycin (Thermo Fisher Scientific, 15140-122) under standard culture conditions (37°C, 5% CO_2_); medium was refreshed every day. Cells were passaged every 4 days with 0.5 mM EDTA (Invitrogen, AM92606) in PBS without Ca^2+^ and Mg^2+^ (PBS^−/−^; Thermo Fisher Scientific, 10010023). To promote better cell survival, 2 µM ROCK inhibitor Thiazovivin (Sigma-Aldrich^®^, SML1045) was added for 24 h after passaging. Cells were differentiated according to differentiation protocols described below.

### Cardiac differentiation

The cardiac differentiation protocol was adapted from well-established protocols (Mendjan et al., 2014; Hofbauer et al., 2021; Zawada et al., 2023). Briefly, pEPSCs were passaged using TrypLE™ and were pre-plated on a gelatinized 6-well tissue culture plates in pEPSCM for 30 min at 37°C and 5% CO_2_ to remove SNL76/7 feeder cells. Thereafter, 240,000 pEPSCs were seeded per well of a 24-well plate in pEPSCM containing 5 μM Y-27632 (day −1). Differentiation was initiated (day 0) by changing the EPSCM to CDM-BSA medium (CDM-BSA: 1:1 DMEM/F-12 with Glutamax (Thermo Fisher Scientific, 31331028) and IMDM (Thermo Fisher Scientific, 21980032) containing 0.1 g/mL BSA (Sigma-Aldrich^®^, A9647), 30 mg/mL transferrin (Sigma-Aldrich^®^, T1147), 1% chemically defined lipid concentrate (Thermo Fisher Scientific, 11905031), ∼0.46 mM (0.004%) of monothioglycerol (Sigma-Aldrich^®^, M6145), supplemented with 10 ng/mL BMP4 (R&D, 314-BP), 1.5 μM CHIR99021 (R&D, 4423), 50 ng/mL Activin A (Sigma-Aldrich^®^, SRP3003), 30 ng/mL bFGF (R&D, 233-FB) and 5 μM LY 294002 hydrochloride (R&D, 1130). Thereafter medium was replaced with CDM-Meso medium [CDM-BSA supplemented with 10 ng/mL BMP4, 8 ng/mL bFGF, 10 μg/mL insulin (Sigma-Aldrich^®^, 11376497001), 5 μM IWP2 (Stemcell Technologies, 72122) and 0.5 μM retinoic acid (RA; Sigma-Aldrich^®^, R2625)] for 4 days with medium change every 24 h. Subsequently, the medium was replaced with CDM-Myo medium (CDM-BSA medium supplemented with 10 ng/mL BMP4, 8 ng/mL bFGF, and 10 μg/mL insulin) for 2 days with medium change every 24 h. The cardiomyocytes were cultured in CDM-Maintenance medium (CDM-BSA supplemented with 10 μg/mL insulin). The medium was replaced every second day. Human ESCs were differentiated into CPCs as described in Zawada et al. (2023).

### Cardiomyocytes dissociation and replating

Five days before dissociation, maintenance medium was changed to EB2 medium consisting of DMEM/F12 (Thermo Fisher Scientific, 21331-020) supplemented with 2% fetal bovine serum (FBS; Sigma-Aldrich^®^, F7524, batch 044M3395), 1% L-glutamine (Thermo Fisher Scientific, 25030-081), 1% non-essential amino acids (Thermo Fisher Scientific, 11140-050), 0.5% penicillin/streptomycin and 0.1 mM beta-mercaptoethanol. Cells were subjected to papain-based dissociation as described by Fischer et al. (2018). Briefly, cells were incubated with papain solution (Worthington Biochemical Corporation, LS003124) for 20 min at 37°C. The enzymatic reaction was stopped with 1 mg/mL trypsin inhibitor (Sigma-Aldrich^®^, T9253) in PBS^−/−^. Cells were reseeded at a density of 100,000-120,000 cells on 12-well chamber slides (Ibidi, 81201) coated with 2 μg/cm^2^ fibronectin (Sigma-Aldrich^®^, F1141) for immunofluorescence analysis or at a density of 50,000-70,000 cells in 3.5 cm glass-bottom cell culture microdishes (MatTek Corporation, P35G-1.5 14-C) coated with 2 μg/cm^2^ fibronectin for calcium and action potential analysis. Cells were incubated for 24 h in EB20 medium consisting of DMEM/F12, 20% FBS, 1% L-glutamine, 1% non-essential amino acids, 0.5% penicillin/streptomycin and 0.1 mM beta-mercaptoethanol. The next day media was changed to EB2 and changed daily till day 65.

### Epicardial and endothelial differentiation of CPCs

Porcine EPSCs were differentiated into epicardial cells as previously described (Bao et al., 2017b; Zawada et al., 2023). On day 6, pEPSC-derived CPCs were detached with Accutase (Thermo Fisher Scientific, A1110501) at 37°C for 5 min and seeded in a density of 20,000 cells/cm^2^ onto 0.1% gelatin-coated wells of 12-well chamber slide or 48-well plates for long-term culture in LaSR medium (Advanced DMEM/ F12 containing Glutamax (Thermo Fisher Scientific, 12634010), 0.1 mg/mL ascorbic acid (Sigma-Aldrich^®^, A5960) and 0.5% penicillin/streptomycin) supplemented with 1% FBS and 10 μM of Y- 27632 for 24 h. On days 7 and 8, the medium was replaced with LaSR medium containing 3 µM CHIR99021. From day 9 to day 12 LaSR medium without additional supplementation was replaced every day. From day 12, epicardial cells were maintained in LaSR medium containing 2 µM SB431542 (R&D, 1614/1) to prevent spontaneous differentiation. For long-term maintenance, cells were passaged 1:4 onto gelatin-coated plates in LaSR medium supplemented with 2 µM SB431542. hESCs were differentiated as described in Zawada et al. (2023).

For endothelial differentiation modified protocol described by Wei et al. (2020) was used. Briefly, d4.5 CPCs were seeded in a density of 20,000 cells/cm^2^ on 12-well chamber slides in LaSR medium containing 50 ng/mL VEGF (R&D, 293-VE-010) and 25 ng/mL BMP4 (R&D, 314-BP). After 8 days, cells were fixed for immunofluorescence staining.

### Differentiation of epicardial cells into ECs, SMCs and cardiac FBs

Porcine EPSC-derived epicardial cells were differentiated into SMCs and FBs as previously described by Bao et al. (2017b). On day −1, pEPSCs were dissociated with Accutase and reseeded onto gelatin-coated plates at a density of 30,000 cells/cm^2^ in LaSR medium containing 5 µM Y27632. From day 0 to day 8, medium was replaced every day with LaSR medium containing 5 ng/mL TGF-β1 (R&D, 7754-BH-005) for SMCs differentiation or with 10 ng/mL bFGF (R&D, 233-FB-025/CF) for FBs differentiation. Epicardial cells were differentiated into ECs as previously described by Bao et al. (2017a) with some modifications. After maintenance in LaSR medium containing 0.5 μM A83-01 for several passages, confluent pEPSC-derived epicardial cells were reseeded at a density of 40,000 cells/cm^2^ on 12-well chamber slides in EGM-2 medium (Lonza, CC-3162) supplemented with 100 ng/mL VEGF. After 8 days cells were fixed for immunostaining.

### RNA isolation, reverse transcription PCR (RT-PCR), and quantitative real-time PCR (qRT-PCR)

Total RNA was isolated from cells using the Absolutely RNA Microprep kit (Agilent, 400805) following manufacturer’s instructions. cDNA was prepared using the High Capacity cDNA RT kit (Applied Biosystems, 4368814) according to the manufacturer’s instructions, and a FlexCycler2 PCR thermal cycler (Analytik Jena, Germany). For long-term storage, RNA was kept at −80°C and cDNA at −20°C. Quantitative real-time PCR (qRT-PCR) was performed using the Power SYBR Green PCR Master Mix (Applied Biosystems, 4367659), 1 µL cDNA, and the gene-specific primers (Table 1). Reactions were run on a 7,500 Fast Real-Time PCR instrument (Applied Biosystems, Germany). The mRNA expression levels of genes of interest were quantified relative to *GAPDH* expression using the ΔCt method.

**TABLE 1:** Quantitative RT (qRT)-PCR primers used for gene expression analysis of porcine cardiac and epicardial cells.

Primer name	Forward sequence	Reverse sequence	References
*ALDH1A2*	AGC​TCT​GTG​CTG​TGG​CAA​TAC	AAA​GCC​AGC​CTC​CTT​GAT​G	
*BNC2*	CTG​AGG​ACT​TGC​GAT​CAG​TGT	GTA​TAG​GTG​CTG​CGT​GCT​GA	
*TBXT*	TCA​AGG​AGC​TCA​CCA​ACG​A	AGA​CAC​GTT​CAC​CTT​CAG​CAC	
*EOMES*	ACT​CCC​ATG​GAC​CTC​CAG​AA	TCG​CTT​ACA​AGC​ACT​GGT​GT	Zhi et al. (2022)
*GAPDH*	CTC​AAC​GGG​AAG​CTC​ACT​GG	CAT​TGT​CGT​ACG​AGG​AAA​TGA​GC	Gao et al. (2019)
*ID2*	TCG​CAC​CCC​ACT​ATT​GTC​AG	TTC​AGA​AGC​CTG​CAA​GGA​CA	Wei et al. (2020)
*ISL1*	CAC​TGT​GGA​CAT​TAC​TCC​CTG​TT	AAC​CAA​CAC​ATA​GGG​AAA​TCA​GAC	
*KDR*	GAG​CCC​CTG​ATT​ACA​CCA​CC	GCA​GAT​ACT​GAC​TGA​TTC​CTG​CT	Wei et al. (2020)
*MESP1*	CGT​CTT​GGG​GGT​CTC​CTT​CTG	GGG​GCC​AAT​ATT​CCA​CCG​TC	Wei et al. (2020)
*MYH6*	TCC​ATC​TCT​GAC​AAC​GCC​TA	TGG​CGA​AGT​ACT​GGA​TGA​CA	
*MYH7*	AAG​GCC​AAG​ATT​TTG​TCT​CG	CTT​GTC​GAA​CTT​GGG​TGG​AT	
*MYL2*	GGG​ACA​CCT​TTG​CTG​CTC​T	ATT​GGA​CCT​GGA​GCT​TCC​TT	
*MYL7*	ATG​GCA​TCA​TCT​GCA​AGT​CA	AAC​GTG​AGG​AAG​ACG​GTG​AA	
*NKX2.5*	CCC​TCG​AGC​CGA​TAA​GAA​AG	ACCTGTGCCTGCGAAAAG	Das et al. (2020)
*SEMA3D*	CAC​GCT​GTT​TCT​TCC​AGT​CA	CAG​CTA​TTT​GAA​AGC​AGC​AAG​TC	
*TBX1*	GTG​AAG​AAG​AAC​GCG​AAG​GT	ATG​CCG​AAA​AGC​TTC​ACT​TG	
*TBX5*	CAC​GAA​GTG​GGC​ACA​GAA​A	TTT​GGG​ATT​AAG​GCC​AGT​CA	
*TBX18*	TTC​ACT​ACG​GAC​TCT​CAC​CTT​TG	CAT​TCC​CAG​AAC​CTT​GGA​GTA​A	
*TCF21*	CAA​CGA​CAA​GTA​CGA​GAA​TGG​TT	TCA​GGT​CAC​TTT​CGG​GTT​TC	
*TNNT2*	CCG​GAA​GAA​GAA​GGC​TCT​GT	CCG​TCT​GCC​TCT​TTC​CAC​T	
*WNT5A*	CGC​GAA​GAC​AGG​CAT​CAA​AG	CCT​ATC​TGC​ATG​ACC​CTG​CC	
*WT1*	GTC​TAC​GGA​TGC​CAC​ACC​TC	AAG​CTG​GGA​GGT​CAT​TTG​GT	

### Immunofluorescence staining of cells

For immunostaining, cells were washed with PBS with Ca^2+^ and Mg^2+^ (PBS^+/+^; Sigma-Aldrich^®^, D8662) and fixed with 4% PFA (Sigma-Aldrich^®^, 158127) for 15 min at room temperature (RT). After washing three times with PBS^+/+^, cells were blocked for 1 h at RT in 0.1% Triton X-100 (Sigma-Aldrich^®^, X100) in PBS^+/+^ containing 3% BSA. Appropriate primary antibodies (Table 2) were then added at the indicated dilutions in PBS^+/+^ containing 0.1% Triton-X-100 and 0.5% BSA and incubated overnight at 4°C. After washing with 0.1% Triton-X-100 in PBS^+/+^, samples were incubated with appropriate secondary antibodies (Table 3) diluted in 0.1% Triton-X-100 PBS containing 0.5% BSA for 1 h at RT. After three washing steps with 0.1% Triton-X-100 in PBS^+/+^, Hoechst 33258 (Sigma-Aldrich^®^, 94403) was added at a final concentration of 5 µg/mL in PBS^+/+^ for 15 min at RT. After washing once with PBS^+/+^, slides with cells were then covered with fluorescence mounting medium (Dako, S3023) and coverslip and stored at 4°C until imaging.

**TABLE 2:** Primary antibodies used for immunostaining.

Target	Host species	Manufacturer	Catalog number	Concentration
α-SMA	Mouse	Sigma-Aldrich^®^	A2547	1:100
ALDH1A2	Rabbit	Abcam	ab96060	1:100
BNC1	Rabbit	Sigma-Aldrich^®^	HPA 063183	1:100
TBXT	Rabbit	R&D Systems	MAB20851-100	1:100
CALPONIN	Mouse	Abcam	AB700	1:100
Cardiac TROPONIN T (cTNT)	Rabbit	Abcam	ab92546	1:500
CD31	Mouse	Bio-Rad	MCA1746GA	1:50
EOMES	Rabbit	Abcam	ab23345	1:100
ISL1	Mouse	Developmental Studies Hybridoma Bank	39.4D5	1:100
KDR/FLK-1/VEGFR2	Rabbit	Cell Signaling	2479	1:100
KRT18 (CK18)	Mouse	Abcam	ab668	1:100
MLC2a	Mouse	Synaptic systems	311 011	1:100
MLC2v	Rabbit	ProteinTech	10906-1-AP	1:100
NANOG	Rabbit	Peprotech	500-p236	1:100
NKX2.5	Rabbit	Invitrogen	PA5-49431	1:100
OCT4	Mouse	Santa Cruz Biotechnology	sc-365509	1:100
SOX2	Goat	R&D Systems	AF2018	1:100
SSEA1	Mouse	Stemcell Technologies	60060AD.1	1:100
SSEA4	Mouse	Stemcell Technologies	60062FI.1	1:100
TBX5	Rabbit	Sigma-Aldrich^®^	HPA064683	1:100
VE-CADHERIN	Mouse	Invitrogen	14-1449-82	1:100
VIMENTIN	Chicken	Abcam	ab24525	1:400
WT1	Rabbit	Abcam	ab89901	1:100
ZO-1	Mouse	Thermo Fischer	33-91010	1:100

**TABLE 3:** Secondary antibodies used for immunostaining.

Target species	Host species	Conjugate	Manufacturer	Catalog number	Concentration
Chicken	Goat	Alexa Fluor 594	Invitrogen	A11042	1:500
Goat	Donkey	Alexa Fluor 647	Invitrogen	A32849	1:500
Mouse	Goat	Alexa Fluor 594	Invitrogen	A11005	1:500
Mouse	Goat	Alexa Fluor 488	Invitrogen	A11001	1:500
Mouse	Goat	Alexa Fluor 647	Invitrogen	A21235	1:500
Rabbit	Goat	Alexa Fluor 488	Invitrogen	A11008	1:500
Rabbit	Goat	Alexa Fluor 647	Invitrogen	A32733	1:500

### Microscopy and image analysis

Stained cells were imaged using confocal laser scanning microscope (TCS SP8; Leica Microsystems, Wetzlar, Germany). Images were acquired and processed using the Leica Application Suite X software (v3.5.7.23225). To quantify the relative distribution of CM subtypes, the number of ventricular (MLC2v^+^), immature ventricular (MLC2v^+^/a^+^) and atrial CMs (MLC2a^+^) was counted using ImageJ (National Institutes of Health).

### Flow cytometry analysis

At day 30 of cardiac differentiation, cells were detached using papain-based dissociation as previously described (Fischer et al., 2018; Zawada et al., 2023). 2 × 10^6^ cells per sample were fixed with 4% PFA for 7 min at RT. After washing three times for 5 min with PBS^+/+^, cells were permeabilized/blocked with 0.1% Triton-X-100, 10% FCS, for 1 h at RT. The primary antibody for cardiac Troponin T (Table 2) was diluted in 1% FCS, with 0.1% Triton-X-100 in PBS^+/+^, and incubated with cells overnight at 4°C. After washing three times for 15 min with PBS^+/+^ containing 0.1% Triton-X-100, an appropriate secondary antibody diluted in 1% FCS in PBS^+/+^ with 0.1% Triton-X-100 was added for 1 h at RT (Table 3). After repeating the previous washing steps with PBS^+/+^ containing 0.1% Triton-X-100 cells were re-suspended in 100 µL PBS^+/+^ supplemented with 2% FCS. Cells were then filtered with a 40 μm filter (Sartorius, 16555-K) and subjected to flow cytometry analysis on Gallios (Beckman Coulter). Data were analyzed using Kaluza software (Beckman Coulter). No-primary antibody, no-secondary antibody, IgG antibody and undifferentiated pEPSCs were performed as controls.

### Calcium imaging

For calcium imaging, day 65 pEPSC-CMs were incubated with calcium indicator Fluo-4 AM (Thermo Fisher, F14201) at a concentration of 1 μM in Tyrode’s solution supplemented with Ca^2+^ as previously described (Moretti et al., 2020). Briefly, field stimulation electrodes (RC-37FS, Warner Instruments, Hamden, CT, United States) were connected to a stimulus generator (HSE Stimulator P, Hugo Sachs Elektronik, March-Hugstetten, Germany) providing depolarizing pulses (50 V, 5 ms duration) at the frequencies indicated. ImageJ (National Institutes of Health, Bethesda, MD) was used to quantify fluorescence over single cells and background regions. Thereafter, analysis was performed in RStudio using custom-written scripts (RStudio, 2020). After subtraction of background fluorescence, the time course of Fluo-4 fluorescence was normalized to the initial value (F/F0). After manual selection of the starting points and peaks of the calcium transients, the transient duration at 50% decay (TD_50_) and 90% decay (TD_90_) was automatically determined by the script. The amplitude of calcium transients was calculated by subtracting the basal fluorescence value from the peak value.

### Optical action potential measurements

For optical action potential imaging a genetically encoded Förster resonance energy transfer (FRET)-mediated membrane potential sensor (voltage-sensitive fluorescent protein, VSFP) was used as previously described (Chen et al., 2017). Briefly, day 58 porcine CMs were reseeded on a 3.5 cm glass-bottom cell culture dishes (MatTek Life Science) using papain-based dissociation and 2 days later transduced with a lentiviral vector encoding the VSFP sensor under the control of the ubiquitous phosphoglycerate kinase 1 (PGK) promoter. Five days after infection, CMs were incubated with Tyrode’s solution and subjected to imaging at 100 frames per second on an inverted epifluorescence microscope (DMI6000B, Leica Microsystems) equipped with a Zyla V sCMOS camera (Andor Technology). Electrical stimulation was performed at 0.5 Hz using field stimulation electrodes as described above. The VSFP was excited at 480 nm, and the emitted GFP and RFP fluorescence signals were separated using an image splitter (OptoSplit II, Caim Research) equipped with CAIRN HQ535.50 566DCXR E570LP filters (Chen et al., 2017; Goedel et al., 2018). The fluorescence over cells and over background regions was quantified in GFP and RFP channels using ImageJ (National Institutes of Health). Custom-written scripts were applied for further analysis in RStudio Team (2020). After background correction, the RFP/GFP ratio corresponding to APs was derived. Cardiomyocytes based on their action potentials were classified into 2 groups: Ventricular-like cardiomyocytes (V-CMs), and immature ventricular-like cardiomyocytes (iV-CMs) based on APD_90_/_50_ ratio. iV-CMs = APD_90_/APD_50_ ratio between 1.4 and 1.8. V-CMs = APD_90_/APD_50_ ratio between 1.0 and 1.4.

### Porcine *in vivo* embryos

Porcine embryos were obtained at ED13, ED14, ED15, ED17, and ED19 of gestation following insemination on two subsequent days. The sows were slaughtered and the uterus was excised and flushed with PBS^−/−^. Collected embryos (11-16 embryos per stage) were fixed in 4% PFA. After washing with PBS^+/+^, the embryos were subjected to a sucrose gradient (5–20%) and embedded in a 1:1 mixture of Tissue-Tek O.C.T. (Sakura, 4583) and 20% sucrose (Sigma-Aldrich^®^, S9378). Samples were frozen in a bath of 2-methylbutane (Sigma-Aldrich^®^, M32631) with liquid nitrogen and stored at −80°C or directly cryo-sectioned into 8 µm-thick sections using cryotome (Microm HM560, Thermo Fisher). Sections were collected onto polylysine-coated slides and stored at −80°C until staining.

### Whole embryo culture *ex utero*

Porcine embryos at ED14 were dissected from uterus and transferred to culture dish with prewarmed PBS^−/−^. Approximately 2 cm of chorioamniotic membrane attached to both ends of each embryo were kept. Immediately after dissection, a single intact embryo was transferred into one glass culture bottle (B.T.C. Engineering, Cullum Starr Precision Engineering Ltd.) in a pre-equilibrated culture medium containing 50% porcine serum (prepared in-house) and 50% DMEM (Thermo Fisher Scientific, 31966). The bottles were placed on a rotating incubator (B.T.C. Engineering) and the complete medium was changed every 24 h. At the end of the culture, embryos were removed from the yolk sac, allantois, and amnion and evaluated in terms of morphological development. Subsequently, embryos were fixed with 4% PFA and processed for cryopreservation as described above.

### Immunofluorescence staining of cryosections

Tissue slides were re-fixed with 3.7% formaldehyde for 15 min at RT. After washing three times with 0.05% Tween-20 in PBS^+/+^ (PBST), sections were permeabilized with 0.1% TritonX-100 for 10 min at RT. After washing briefly with PBST sections were blocked with 10% FBS in PBST for 1 h at RT. The slides were incubated with primary antibodies (Table 2) in PBS^+/+^ containing 1% FBS and 0.1% TritonX-100 at 4°C overnight. Subsequently, sections were washed three times with PBST, incubated with appropriate secondary antibodies (Table 3) for 1 h at RT. After washing three times with PBST, samples were counterstained with Hoechst 33258 at a final concentration of 5 µg/mL in PBS^+/+^ for 15 min at RT. After a final wash with PBS^+/+^, sections were covered with fluorescence mounting medium (Dako, S3023) and stored at 4°C until imaging with confocal laser scanning microscopy (TCS SP8; Leica Microsystems, Wetzlar, Germany) or Leica THUNDER system.

### Statistical analysis

Statistical analyses were performed with GraphPad Prism 9.1.0 (La Jolla California, United States). Bar graphs indicate the mean ± SEM with all data points displayed separately. For calcium imaging, a *p*-value < 0.05 was considered statistically significant.

## Results

### Anatomical and molecular profile of porcine heart development

**FIGURE 1:**
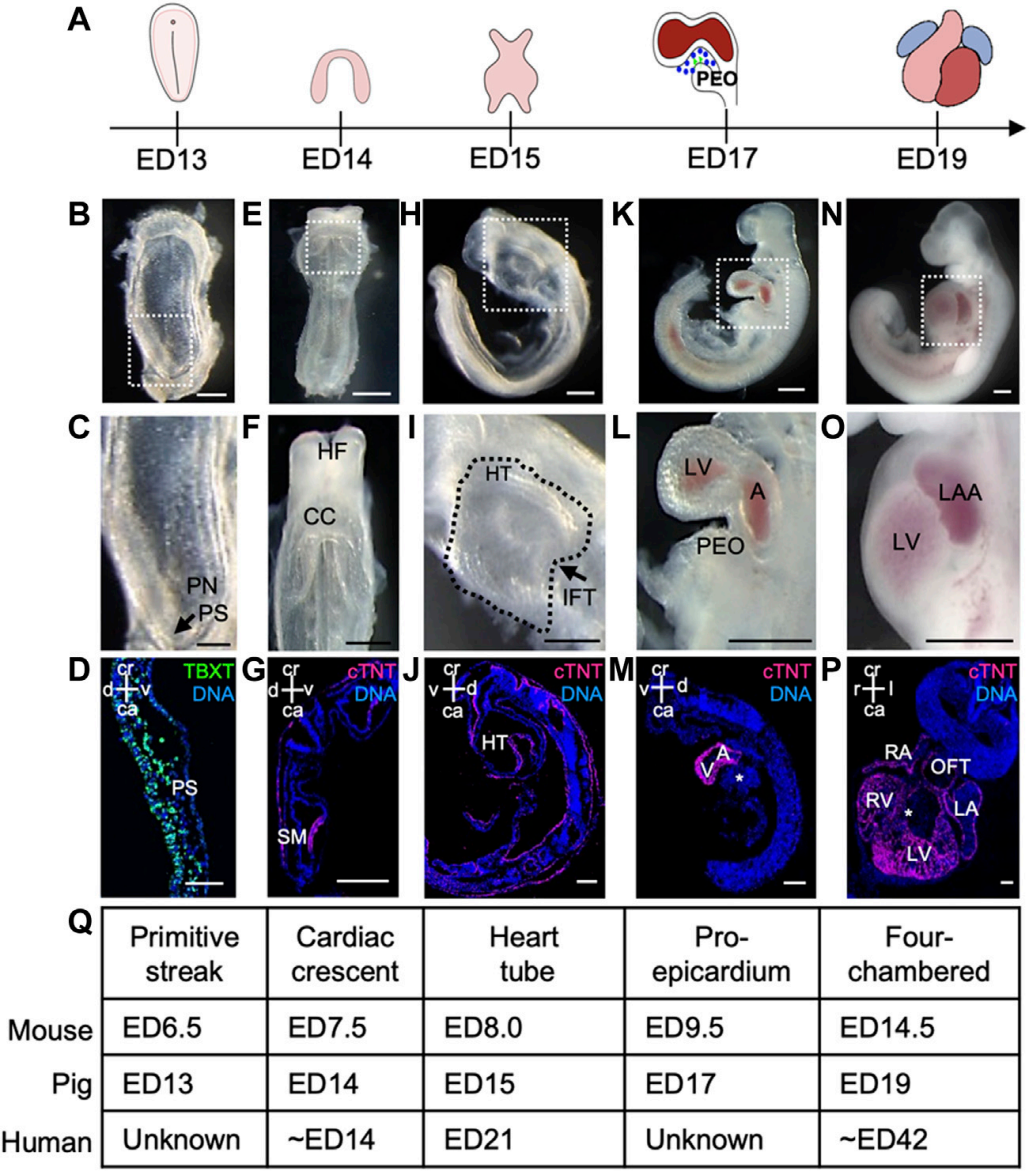
Porcine heart development from ED13 to ED19. **(A)** Graphical representation of different stages of porcine cardiac development. **(B, C)** Frontal view of ED13 porcine embryo displaying primitive streak (arrow). The boxed region in **(B)** is shown at higher magnification in **(C)**. **(D)** Representative image of sagittal section of ED13 embryo after immunofluorescence analysis of TBXT (green). Nuclei were labeled with Hoechst 33528 (blue). **(E, F)** Frontal view of ED14 embryo depicting the cardiac crescent. The boxed region in **(E)** is shown at higher magnification in **(F)**. **(G)** Representative image of transverse section of ED14 embryo after immunofluorescence analysis of cTNT (magenta). Nuclei were labeled with Hoechst 33528 (blue). **(H, I)** Left view of ED15 embryo showing the primitive heart tube. The boxed region in **(H)** is shown at higher magnification in **(I)**. **(J)** Representative image of sagittal section of ED15 embryo after immunofluorescence analysis of cTNT (magenta). Nuclei were labeled with Hoechst 33528 (blue). **(K, L)** Left view of ED17 embryo displaying the proepicardium, common atrium, and left ventricle. The boxed region in **(K)** is shown at higher magnification in **(L)**. **(M)** Representative image of sagittal section of ED17 embryo after immunofluorescence analysis of cTNT (magenta). Nuclei were labeled with Hoechst 33528 (blue). **(N, O)** Left view of ED19 embryo with four-chambered heart. The boxed region in **(N)** is shown at higher magnification in **(O)**. **(P)** Representative image of frontal section of ED19 embryo after immunofluorescence analysis of cTNT (magenta). Nuclei were labeled with Hoechst 33528 (blue). **(Q)** Table summarizing major events during heart development in pig, mouse and human. A, atrium; ca, caudal; CC, cardiac crescent; cr, cranial; d, dorsal; HT, heart tube; IFT, inflow tract; l, left; LA, left atrium; LAA, left atrial appendage; LV, left ventricle; OFT, outflow tract; PEO, proepicardial organ; PN, primitive node; PS, primitive streak; r, right; SM, splanchnic mesoderm; v, ventral; V, ventricle. Asterix indicates interventricular septum. Scale bars for bright-field images 1 mm, for immunostaining images: 10 μm.

Porcine preclinical models are now considered the gold standard for studying congenital heart diseases (CHDs) (Buijtendijk et al., 2020; Gabriel et al., 2021), as numerous recent studies have highlighted the anatomical and molecular similarities in cardiac development between pigs and humans (Lelovas et al., 2014; Lauschke et al., 2021). However, a detailed characterization of the early stages of porcine cardiogenesis is still lacking. Here, we examined the porcine heart development from the primitive streak stage (ED13) to the four-chambered beating heart (ED19). The embryos were collected at five different time points (ED13, ED14, ED15, ED17, and ED19) (Figure 1A) and subjected to anatomical and immunohistochemical analyses (Figures 1B–P; Supplementary Figure S1).

At ED11-12, the porcine spherical blastocyst starts to elongate and forms a filamentous structure (Hyttel et al., 2011). This process is accompanied by the initiation of gastrulation, establishing the primitive streak. At ED13, the primitive streak was visible at the caudal end of the porcine embryo (Figures 1A–D), which was marked by the expression of mesodermal T-box transcription factor TBXT (BRACHYURY) (Figure 1D) and cardiac mesoderm-specific marker KDR (Supplementary Figures S2A–C) corresponding to the stage ED6.5 in the mouse (Krishnan et al., 2014). The first cells expressing cardiac troponin T (cTNT) were detected at ED14 (ED7.5 in mouse and approximately week 2 of human gestation) (Krishnan et al., 2014; Buijtendijk et al., 2020) in splanchnic mesoderm forming the cardiac crescent (Figures 1E–G, Q). At ED15, a faintly beating linear heart tube could be visualized (ED8.0 in mouse and week 3 of human gestation) (Figures 1H–J, Q), which subsequently underwent looping and around ED17 (ED9.5 in mouse) common atrium and ventricle could be distinguished (Krishnan et al., 2014; Buijtendijk et al., 2020) (Figures 1K–M, Q). Notably, at ED17 an extracardiac cluster of cells was detectable at the base of the venous pole of the embryonic heart resembling the proepicardial organ (PEO) in mouse at ED9.5 (Figures 1L, Q) (Krishnan et al., 2014). By ED19 the embryo and extraembryonic membranes increased in size (Figures 1N, O, Q; Supplementary Figures S1J–N). The heart had developed a four-chambered structure, and the heartbeat became more prominent (Figures 1N–P). The interventricular septum, atrial septum, atrial appendages, compact myocardium, and trabecular myocardium were also clearly visible at ED19 (Figure 1P; Supplementary Figure S1).

Next, we sought to provide a more comprehensive characterization of the porcine cardiac precursors giving rise to the various cardiac lineages. In mouse embryo, three spatially and temporarily distinct populations of cardiac progenitors have been identified and described in detail: the cardiogenic mesoderm cells, which encompasses first and second heart field (FHF and SHF), precursors of the PEO, and cardiac neural crest cells (Brade et al., 2013). Here, we focussed primarily on the cardiogenic mesoderm and PEO (Moretti et al., 2006; Zhou et al., 2008; Buijtendijk et al., 2020).

Within the cardiogenic mesoderm, FHF progenitors reside in the cardiac crescent and form a linear heart tube, which later becomes the left ventricle, whereas SHF precursors are located posteriorly and medially to the FHF and give rise to the outflow tract, right ventricle, a subset of left ventricular cells, and atria (Brade et al., 2013; Paige et al., 2015; Meilhac and Buckingham, 2018; Ivanovitch et al., 2021). NKX2.5 and ISL1 are the key cardiac-specific transcription factors that mark FHF and SHF and play a pivotal role in early heart development (Lyons et al., 1995; Cai et al., 2003; Moretti et al., 2006). While NKX2.5 is expressed in cardiac precursors of both heart fields and differentiated CMs (Lints et al., 1993; Kasahara et al., 1998), ISL1 shows transient expression in FHF and is mainly restricted to the SHF progenitors. Furthermore, it is absent in differentiated states (Moretti et al., 2006).

**FIGURE 2:**
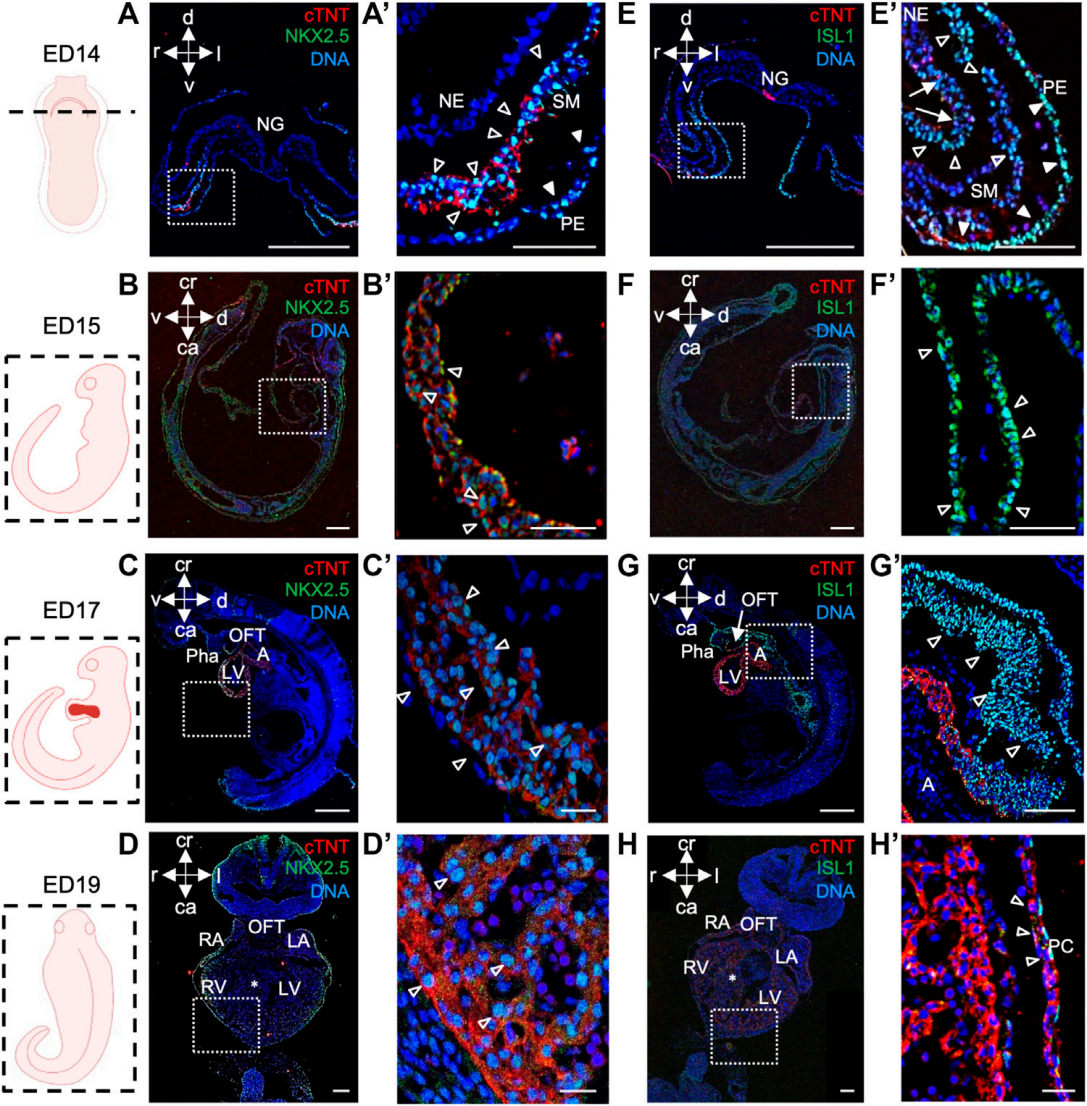
NKX2.5 and ISL1 expression during porcine heart development from ED14 to ED19. **(A–D′)** Representative images of ED14 **(A, A′)**, transverse, ED15 **(B, B′)**, sagittal, ED17 **(C, C′)**, sagittal, and ED19 **(D, D′)**, frontal embryo sections after immunofluorescence analysis of NKX2.5 (green) and cTNT (red). Nuclei were labeled with Hoechst 33528 (blue). At ED14, NKX2.5^+^ cells were detected in the splanchnic mesoderm (empty arrowheads) and pharyngeal endoderm (filled arrowheads) **(A, A′)**. At ED15, ED17, and ED19, NKX2.5 expression was observed in cardiomyocytes of the developing heart (empty arrowheads) **(B′–D′)**. **(E–H′)** Representative images of ED14 **(E, E′)**, transverse, ED15 **(F, F′)**, sagittal, ED17 **(G, G′)**, sagittal, and ED19 **(H, H′)**, frontal embryo sections after immunofluorescence analysis of ISL1 (green) and cTNT (red). Nuclei were labeled with Hoechst 33528 (blue). At ED14, ISL1^+^ cells were found in splanchnic mesoderm (empty arrowheads), pharyngeal endoderm (filled arrowheads), and neuroectoderm (arrows) **(E, E′)**. At ED15, ISL1 expression was detected in SHF (filled arrowheads) and outflow tract (empty arrowheads) of the primitive heart tube **(F, F′)**. At ED17, ISL1 expression was detected in cells of OFT **(G)**, arrow and SHF **(G′)**, empty arrowheads. At ED19, ISL1^+^ cells were sparsely detected in the pericardium (empty arrows) **(H, H′)**. Sections correspond to the position indicated by the plane drawn through the adjacent embryo view. The white boxes indicate a region of higher magnification shown in adjacent panels. A, atrium; ca, caudal; cr, cranial; d, dorsal; IFT, inflow tract; l, left; LA, left atrium; LV, left ventricle; NE, neuroectoderm; NG, neural groove; OFT, outflow tract; Pha, pharyngeal arch; PC, pericardium; r, right; RA, right atrium; RV, right ventricle; SHF, second heart field; SM, splanchnic mesoderm; v, ventral; asterix indicates interventricular septum. Scale bars: 10 µm.

In porcine embryos at ED14, we detected NKX2.5^+^ and ISL1^+^ cells in splanchnic mesoderm, where cardiogenic progenitors reside, and in pharyngeal endoderm (Figures 2A, A′, E, E′). ISL1^+^ cells were also present in neuroectoderm (Figure 2E′). Co-staining of both proteins allowed us to identify a population of NKX2.5^high^/ISL1^low^- and NKX2.5^high^/ISL1^high^-expressing progenitors, resembling FHF and SHF precursors in mouse, respectively (Mommersteeg et al., 2010) (Supplementary Figures S2D–G). At later stages ED15, ED17, and ED19, NKX2.5 was expressed in CMs of the developing heart (Figures 2B–D′), whereas ISL1^+^ cells were detected in SHF and outflow tract (OFT) at ED15 and ED17 (Figures 2F–G′), which is similar to the mouse (Kasahara et al., 1998; Cai et al., 2003). By ED19, no more ISL1^+^ cells could be found within the heart (Figure 2H), although very few cells in the pericardium persisted to express ISL1 (Figure 2H′).

**FIGURE 3:**
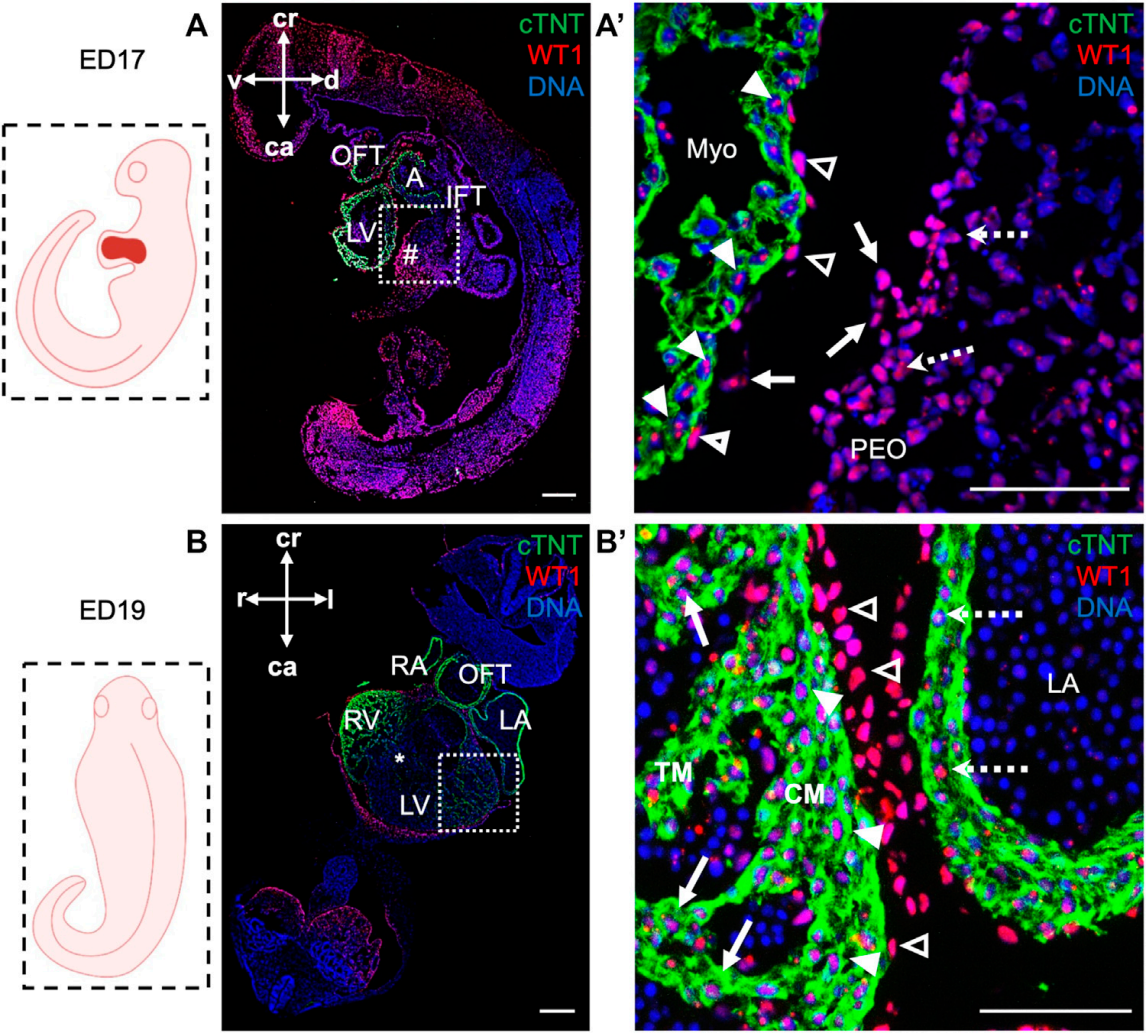
WT1 expression in proepicardium and epicardium of ED17 and ED19 porcine hearts. **(A, A′)** Representative images of sagittal section of ED17 embryo after immunofluorescence analysis of cTNT (green) and WT1 (red) depicting WT1 expression in proepiardial (dashed arrows), in PEO-derived translocating cells (arrows), epicardial (empty arrowheads) cells and cardiomyocytes (filled arrowheads). Nuclei were labeled with Hoechst 33528 (blue). The boxed region in **(A)** is shown at higher magnification in **(A′)**. Scale bars: 10 µm. **(B, B′)** Representative images of frontal section of ED19 embryo after immunofluorescence analysis of cTNT (green) and WT1 (red) showing WT1 expression in epicardial cells (empty arrowheads), ventricular cardiomyocytes in compact (filled arrowheads) and trabecular myocardium (arrows) and in atrial cardiomyocytes (dashed arrows). Nuclei were labeled with Hoechst 33528 (blue). The boxed region in **(B)** is shown at higher magnification in **(B′)**. Scale bars: 10 µm. Sections correspond to the position indicated by the plane drawn through the adjacent embryo view. A, atrium; ca, caudal; CM, compact myocardium; cr, cranial; d, dorsal; l, left; LV, left ventricle; Myo, myocardium; OFT, outflow tract; PEO (#), proepicardial organ; r, right; RV, right ventricle; TM, trabecular myocardium; v, ventral; asterix indicates interventricular septum.

**FIGURE 4:**
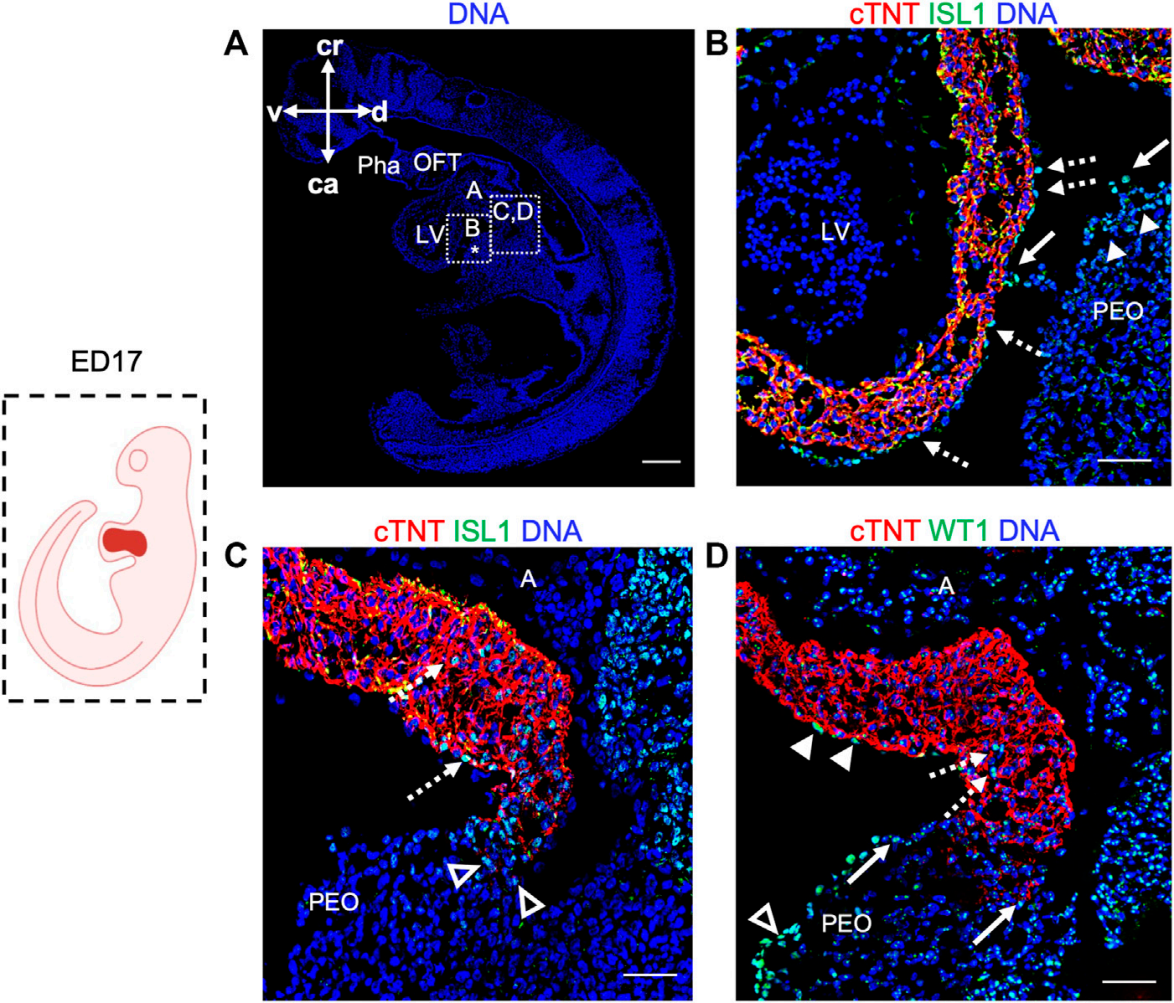
Expression of ISL1 and WT1 in ED17 porcine hearts. **(A)** Representative image of sagittal section of ED17 embryo stained with Hoechst 33528 (blue). The white boxes indicate regions of higher magnification shown in **(B–D) (B–D)** Representative images of sagittal section of ED17 embryo after immunofluorescence analysis of cTNT (red), ISL1 (green), and WT1 (green). ISL1 expression was detected in cells emerging from the proepicardium (filled arrowheads), translocating towards epicardium (arrows), in the newly formed epicardium (dashed arrows) **(B)**, at the junction of the PEO and the base of the atrium (empty arrowheads), and atrium (dashed arrows) **(C)**. WT1^+^ cells were present at the PEO-atrium junction (arrows), atrium (dashed arrows), epicardium (filled arrowheads), and PEO (empty arrowheads). Scale bars: 10 µm. A, atrium; ca, caudal; cr, cranial; d, dorsal; l, left; LV, left ventricle; OFT, outflow tract; PEO (*), proepicardial organ; Pha, pharyngeal arch; v, ventral.

Next, we took advantage of ED17 porcine embryos to examine and further characterize the PEO, which gives rise to the epicardium, the outermost mesothelial layer of the heart (Cao et al., 2020). In mouse, the PEO is a transient extra-cardiac structure that arises at the septum transversum of the venous pole at ED9.0 -ED10.5 and is derived from NKX2.5/ISL1 expressing progenitors. PEO is marked by the expression of several transcription factors, including Wilm’s Tumor 1 (WT1), which continues to be expressed in the epicardium (Wagner et al., 2005). Immunofluorescence analysis of ED17 porcine hearts demonstrated WT1 expression in the cell cluster at the ventro-caudal base of the developing heart corresponding to PEO (Figures 3A, A′). Importantly, we could capture single cells in the PEO translocating across the pericardial cavity and adhering to the myocardial layer of the developing heart (Figure 3A′). At this stage WT1^+^ epicardial cells were sparsely scattered around the heart (Figure 3A′) and by ED19 they uniformly enveloped the myocardium (Figures 3B, B′). Strikingly, few cells emerging from the PEO and attaching to the myocardium also expressed ISL1 (Figures 4A, B), as well as cells at the junction of PEO and base of the atrium (Figure 4C), the area that was positive for WT1 (Figure 4D). This differs from the mouse, where ISL1 is not expressed in these cells at comparable stages (Ruiz-Villalba et al., 2013; Zhuang et al., 2013; Niderla-Bielinska et al., 2019). At ED19, ISL1 expression was lost in the porcine epicardium and became restricted to the pericardium, which was negative for WT1 (Supplementary Figure S3). Interestingly, we observed WT1 expression in the cardiomyocytes of the developing heart at ED17 (Figure 3A′) as well as in the compact and trabecular myocardium and atria at ED19 (Figure 3B′), which is in line with the recent reports describing WT1 expression in cardiomyocytes in mice at ED10.5 (Diaz Del Moral et al., 2021; Wagner et al., 2021).

### *Ex utero* culture of porcine embryos from cardiac crescent to proepicardial organ specification

**FIGURE 5:**
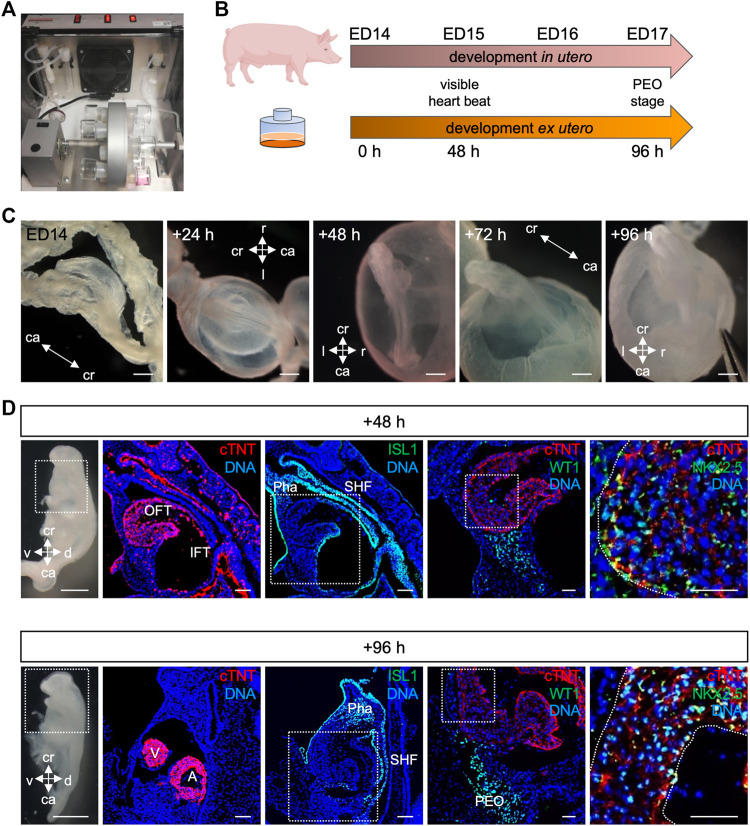
Culture of porcine embryos *ex utero*. **(A)** Picture depicting rotating incubator used for porcine embryo culture *ex utero*. **(B)** Schematic comparison of porcine embryonic development *in utero* and *ex utero* based on two developmental hallmarks (heart beat and PEO formation). **(C)** Bright-field images of embryo at ED14, after 24, 48, 72, and 96 h *ex utero* culture. **(D)** On the left, bright-field images of the dissected *ex utero* cultured embryos after 48 h (top) and 96 h (bottom) are shown. Right beside corresponding representative images of sagittal sections after immunofluorescence analysis for cTNT (red), ISL1 (green), WT1 (green), and NKX2.5 (green). Nuclei were labeled with Hoechst 33528 (blue). The white box indicates a region of higher magnification shown in the adjacent right panel. Scale bars for bright-field images: 500 μm, for immunostaining images: 50 µm. A, atrium; ca, caudal; cr, cranial; d, dorsal; IFT, inflow tract; OFT, outflow tract; Pha, pharyngeal arch; PEO, proepicardial organ; SHF, second heart field; v, ventral; V, ventricle.

Recent studies using *ex utero* culture of mouse embryos have recapitulated *in utero* development and thus opened new possibilities to study mammalian development and disease (Aguilera-Castrejon et al., 2021; Zawada et al., 2023). Here, we report for the first time the culture of porcine embryos at cardiac crescent stage for up to 4 days using a rotating incubator (Figure 5A). We applied the same conditions as for the mouse embryo culture (20% O_2_, 5% CO_2_, 30 rpm, 37°C) (Aguilera-Castrejon et al., 2021) and observed similar development in terms of morphology, initiation of heartbeat and growth compared to the *in utero* situation (Figures 5B, C). Embryos were examined after 24, 48, 72, and 96 h of *ex utero* culture. The beating of the heart was detected after 48 h and persisted at 96 h of culture, although weaker. Morphological and immunohistochemical analyses revealed development of a primitive heart tube within 48 h and PEO within 96 h of culture, resembling ED15 and ED17 embryos *in vivo*, respectively (Figure 5D). At these stages, ISL1 was expressed in the second heart field and NKX2.5 was present in CMs of the developing heart, similarly to the *in utero* counterpart. WT1 could be detected after 48 h of culture and after 96 h WT1 expressing cells were identified underneath the atrioventricular cavity forming the PEO as seen *in vivo*. Together, we could culture porcine embryos from cardiac crescent up to PEO stage, and recapitulate porcine early embryonic development *ex utero*. This system offers new possibilities for genetic manipulation of porcine embryos and for life-monitoring of developing structures.

### Porcine CPCs and CMs derived from pEPSCs express key lineage commitment markers

The availability of functional porcine CPCs and CMs is essential for cardiac disease modeling or testing of autologous cell therapy in the preclinical pig model. However, differentiation of porcine PSCs into the numerous cardiac lineages has not yet been achieved.

**FIGURE 6:**
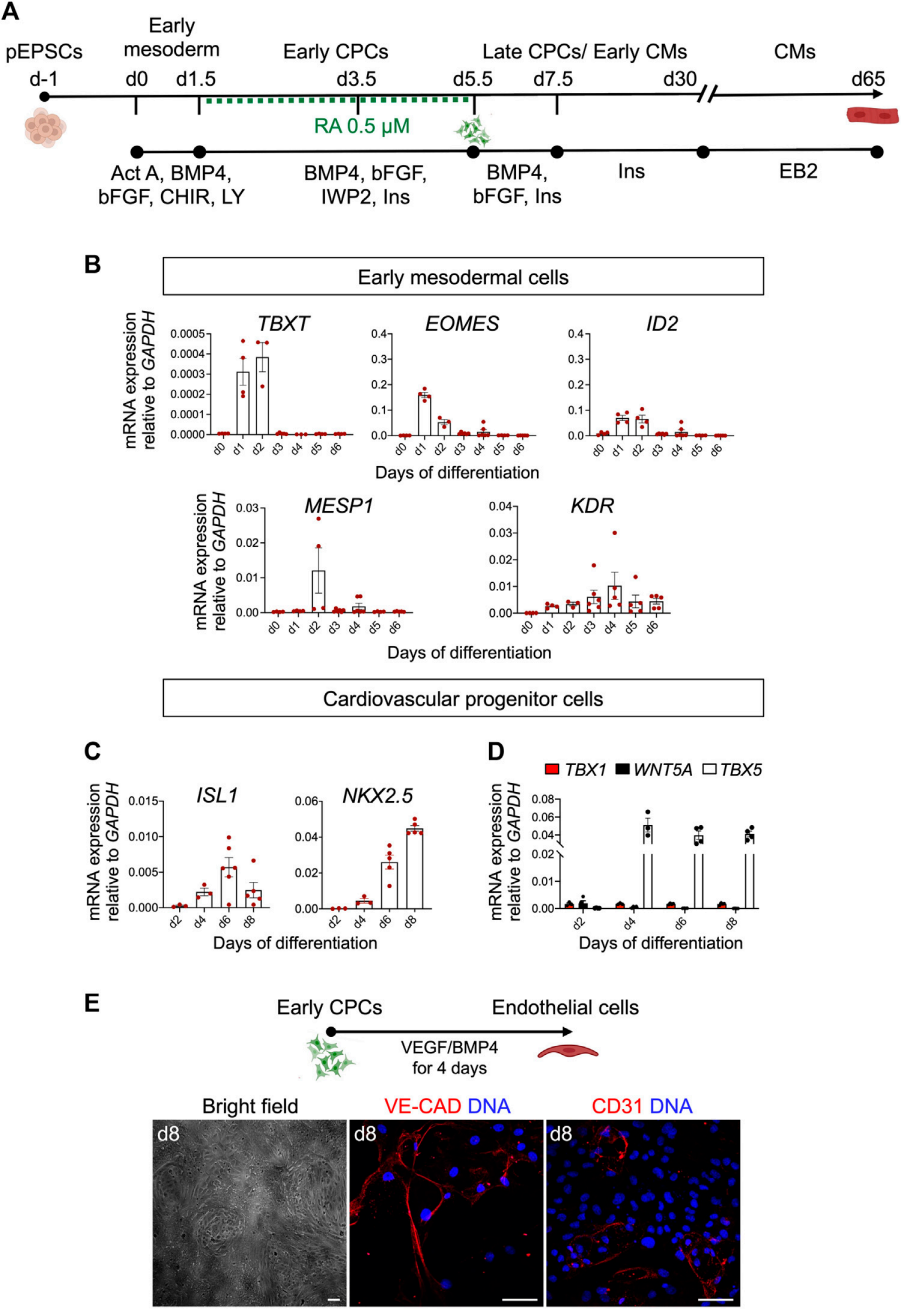
Differentiation of pEPSCs into early mesodermal cells. **(A)** Schematic representation of the protocol used to differentiate porcine expanded potential stem cells (EPSCs) into cardiomyocytes (CMs) through defined steps of early mesoderm and cardiovascular progenitor cells (CPCs). Act A, Activin A; CHIR, CHIR99021, LY, LY-29004, RA, retinoic acid, Ins, Insulin. **(B)** mRNA expression levels of *TBXT*, *EOMES*, *ID2*, *MESP1* and *KDR* relative to *GAPDH* during the first 6 days of cardiac differentiation. Data are mean ± SEM; *n* = 3–6 differentiations. **(C)** mRNA expression level of *ISL1*, *NKX2.5* in CPCs. **(D)** mRNA expression of FHF marker *TBX5* and anterior SHF markers *TBX1* and *WNT5A* relative to *GAPDH* during 8 days of cardiac differentiation. Data are mean ± SEM; *n* = 3–6 differentiations. **(E)** Top panel: Schematic representation of the protocol used to differentiate day 4.5 cardiovascular progenitor cells (CPCs) into endothelial cells (ECs). Bottom panel: Representative bright-field and immunofluorescence images of CPC-derived ECs stained for CD31 (red) and VE-CADHERIN (red) at day 8 of differentiation. Nuclei were labeled with Hoechst 33258 (blue). Scale bars: 50 μm. Images are representative of three independent differentiations.

To investigate whether CMs can be derived from pEPSCs, we utilized a stepwise 2D differentiation protocol for directed differentiation of human PSCs towards CMs using low/mid-dose dosage of retinoic acid (Zawada et al., 2023) (Figure 6A). This protocol is based on temporal control of key cardiogenic signaling pathways, including Activin/Nodal, bone morphogenic protein (BMP), fibroblast growth factor (FGF), Wnt and retinoic acid (RA), recapitulating induction of FHF-like cells and (left) ventricular-like CMs (Zawada et al., 2023).

The differentiation of pEPSCs into CMs progresses through multiple steps of cell-fate determination, and each stage can be captured by the expression of identity-specific marker genes. Porcine EPSCs, which expressed pluripotency markers, including OCT4, NANOG, SOX2, SSEA1 and SSEA4 (Supplementary Figure S4A), differentiated in the first step into primitive streak/early mesoderm-like cells upon activation of Wnt pathway by GSK-3β inhibition using CHIR99021 (CHIR) and phosphatidylinositol 3-kinase inhibitor LY294002 (Ly), and parallel stimulation of FGF and Activin/Nodal pathways by basic FGF (bFGF) and Activin A, respectively. At day 1, we detected expression of *TBXT* (*BRACHYURY)*, indicative of the primitive streak stage *in vivo*, as well as *EOMES*, *ID*2, and *KDR* marking the earliest cardiac mesodermal cells, followed by upregulation of *MESP1* at day 2 (Figure 6B). Subsequent Wnt inhibition from day 1.5 to day 5.5 by IWP2 and supplementation with BMP4, bFGF and RA induced expression of CPCs markers *ISL1*, *NKX2.5* and *TBX5* (Figures 6C, D). Timing and patterns of gene expression corresponded to those described recently by our group during *in vitro* differentiation of hPSCs (Zawada et al., 2023). With the progression of differentiation, we observed a downregulation of *ISL1* and increased expression of *NKX2.5* (Figure 6C). Thereafter we analysed the expression of key FHF marker *TBX5* and anterior SHF markers *TBX1* and *WNT5A*. Notably, we confirmed only an abundant presence of *TBX5* transcripts in the porcine CPCs suggesting FHF-like fate acquisition similar to hPSCs (Figure 6D) (Zawada et al., 2023). Interestingly, porcine CPCs arising at day 4.5 of differentiation had also the potential to differentiate into ECs upon treatment with VEGF and BMP4, as indicated by expression of CD31 and VE-CADHERIN as well as cobblestone-like morphology (Figure 6E).

**FIGURE 7:**
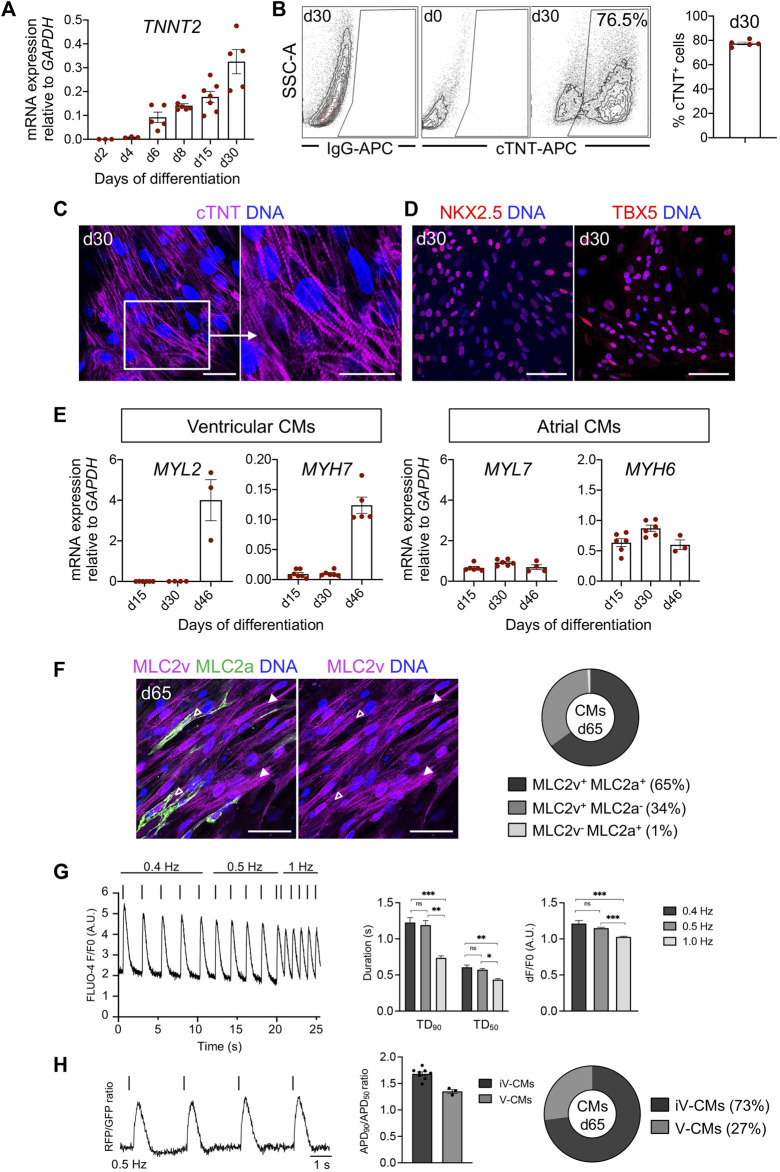
Characteristics of pEPSC-derived CMs. **(A)** mRNA expression level of *TNNT2* relative to *GAPDH* during 30 days of the differentiation. Data are mean ± SEM; *n* = 3–6 differentiations. **(B)** Representative plots of flow cytometry analysis (left) and percentage of cells positive for cTNT at day 30 of differentiation (right); *n* = 4 independent differentiations. **(C)** Representative immunofluorescence images for the cTNT (magenta) in CMs at d30. The white box indicates a region of higher magnification shown in the adjacent right panel. Nuclei were labeled with Hoechst 33258 (blue). Scale bars: 50 μm. *n* = 8 independent differentiations. **(D)** Representative images of immunostaining for NKX2.5 (red), TBX5 (red) in CMs at day 30. Nuclei were labeled with Hoechst 33258 (blue). Scale bars: 50 μm. *n* = 8 independent differentiations. **(E)** mRNA expression level of markers specific for ventricular (*MYL2*, *MYH7*) and atrial (*MYL7*, *MYH6*) CMs relative to *GAPDH*. Data are mean ± SEM, *n* = 3–6 independent experiments. **(F)** Left panel: Representative immunofluorescence images of CMs stained for MLC2v (magenta) and MLC2a (green) at day 65. Immature CMs expressing both MLC2v^+^ and MLC2a^+^ were marked with empty arrows and mature CMs expressing only MLC2v^+^ with filled arrowheads. Nuclei were labeled with Hoechst 33258 (blue). Scale bars: 50 μm, *n* = 4 independent differentiations. Right panel: Percentage of CMs expressing MLC2a^+^ and MLC2v^+^ (65%), MLC2v^+^ (34%) and MLC2a^+^ (1%) at day 65, *N* = 579 cells, *n* = 2 differentiations. **(G)** Left panel: Representative trace of Fluo-4-based intracellular calcium transient of porcine CMs at day 65 with increasing pacing rates (0.4–1 Hz). Middle panel: Ca^2+^ transient duration at 90% and 50% decay (TD_90_ and TD_50_). Right panel: Calcium transient amplitudes. Data are mean ± SEM; *N* = 42 cells, *n* = 3 independent differentiations. *p < 0.05, **p < 0.01, ***p < 0.0001 (Kruskal–Wallis test with a Dunn’s multiple comparisons test). **(H)** Left panel: Representative optical AP traces of porcine CMs transduced with a lentiviral vector encoding PGK-voltage-sensitive fluorescent protein at 0.5 Hz stimulation at day 65. Middle panel: Ratio of APD_90_/APD_50_ at 0.5 Hz. Data are mean ± SEM; *N* = 11 cells, *n* = 2 independent differentiations. Right panel: Percentage of cardiomyocyte subtypes at day 65 of differentiation based on the ratio of APD_90_/APD_50_. iV-CMs immature ventricular cardiomyocytes, V-CMs ventricular cardiomyocytes.

During differentiation, upregulation of *TNNT2* gene was observed from day 6 onward (Figure 7A), which corresponds to the first observed cTNT expression in the porcine cardiac crescent at ED14 (Figure 1G). Importantly, we validated our mRNA results using immunofluorescence analysis showing a similar expression pattern of TBXT, EOMES and KDR (Supplementary Figure S4B). Porcine CPCs were stained positive for ISL1 and early CMs for NKX2.5 (Supplementary Figure S4C). Spontaneously contracting porcine CMs emerged in 80% of differentiation experiments at day 8 and in around 20% of experiments at days 9 and 10 (*n* = 8) (Supplementary Video). Using flow cytometry analyses we detected ∼76% of cTNT^+^ cells at day 30 of differentiation (Figure 7B). The CMs had elongated morphology and showed well-organized sarcomeres, as visualized by immunostaining for cTNT at day 30 (Figure 7C). Furthermore, immunofluorescence analysis revealed that they expressed TBX5 and NKX2.5 at day 30 (Figure 7D).

Next, we examined the expression of cardiac subtype-specific markers during differentiation. Transcripts for myosin light chain 2v (*MYL2*) and myosin heavy chain 7 (*MYH7*)—markers that are specific for ventricular CMs—increased during differentiation, whereas expression of markers typical of atrial or immature CMs, such as myosin light chain 2a (*MYL7*) and myosin heavy chain 6 (*MYH6*) remained constant over time (Figure 7E).

Immunofluorescence analysis for the ventricular and atrial-specific myosin light chain isoforms (MLC2v and MLC2a) indicated that most of the CMs at day 65 (99%) were positive for MLC2v, implying a ventricular-like identity. Many of them still expressed MLC2a and represented immature ventricular CMs. We observed only around 1% of MLC2a^+^/MLC2v^−^ CMs, likely corresponding to an atrial population (Figure 7F).

We further validated the functionality of the pEPSC-derived CMs at day 65 using calcium and optical action potential (AP) imaging. The porcine CMs responded to electrical stimulation and demonstrated a reduction in Ca^2+^ transient duration at 50% and 90% decay (TD_50_ and TD_90_) as well as in calcium transient amplitude at increasing pacing frequencies (0.4 Hz–1.0 Hz), indicative of normal Ca^2+^ handling (Figure 7G). Optical AP traces obtained from porcine CMs were comparable to those previously recorded from human CMs (Chen et al., 2017). CMs demonstrated a ratio of AP duration at 90% and 50% repolarization (APD_90_ and APD_50_) typical of the ventricular CM lineage (APD_90_/APD_50_ = 1.0–1.8) (Figure 7H). In line with immunofluorescence results, AP measurements indicated a primarily immature ventricular-like profile of porcine CMs (Figures 7F, H).

In summary, we could demonstrate that porcine CPCs in our differentiation conditions acquired a FHF-like fate and gave rise to ventricular-like CMs similar to hPSCs (Zawada et al., 2023). Overall, these findings confirm that the developmental pathways that take place in the embryonic porcine heart *in vivo* can be replicated during cardiogenesis *in vitro* using pEPSCs.

### Differentiation and long-term maintenance of pEPSC-derived epicardial cells

The epicardium, as an outer mesothelial layer of the heart, plays a crucial role during heart development by providing the majority of cardiac FBs and vascular SMCs. It is also essential for myocardial growth and repair, making epicardial cells a relevant population for preclinical testing (Cai et al., 2003; Cai et al., 2008; Bao et al., 2016).

**FIGURE 8:**
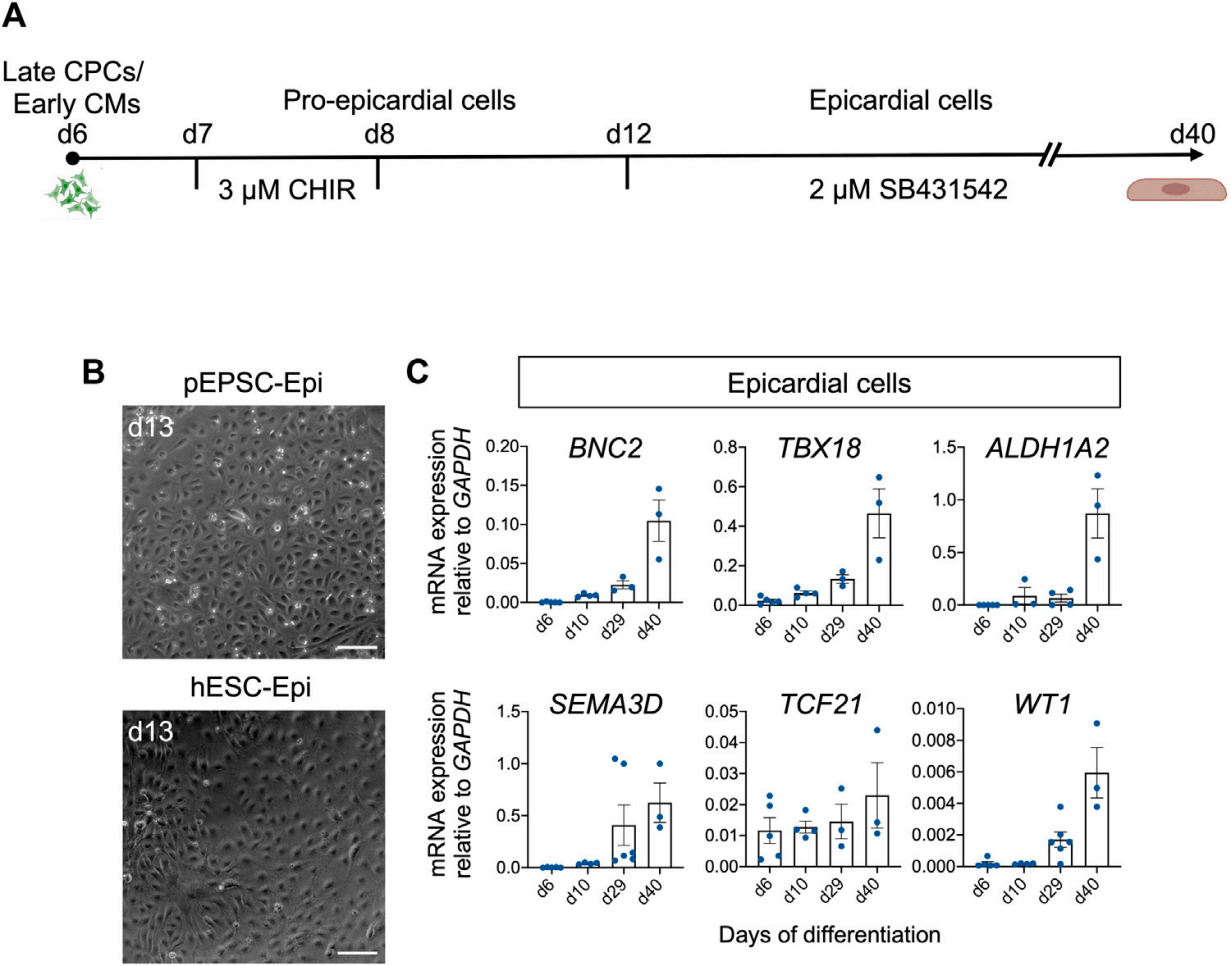
Differentiation of pEPSCs and hESCs into epicardial cells. **(A)** Graphical representation of the protocol used to differentiate pEPSCs and hESCs into epicardial cells. CHIR: CHIR99021. CPCs: cardiovascular progenitors, CMs: cardiomyocytes. **(B)** Representative bright-field images of hESC- and pEPSC-derived epicardial cells displaying typical cobblestone morphology at day 13 of differentiation. Scale bars: 100 μm. hESC-Epi: human embryonic stem cell-derived epicardial cells; pEPSC-Epi: porcine expanded potential stem cell-derived epicardial cells. **(C)** mRNA expression levels of markers specific for epicardial cells *BNC2*, *TBX18*, *ALDH1A2*, *SEMA3D*, *TCF21* and *WT1* relative to *GAPDH* at days 6, 10, 29, and 40 of differentiation. Data are mean ± SEM; *n* = 3–6 differentiations.

**FIGURE 9:**
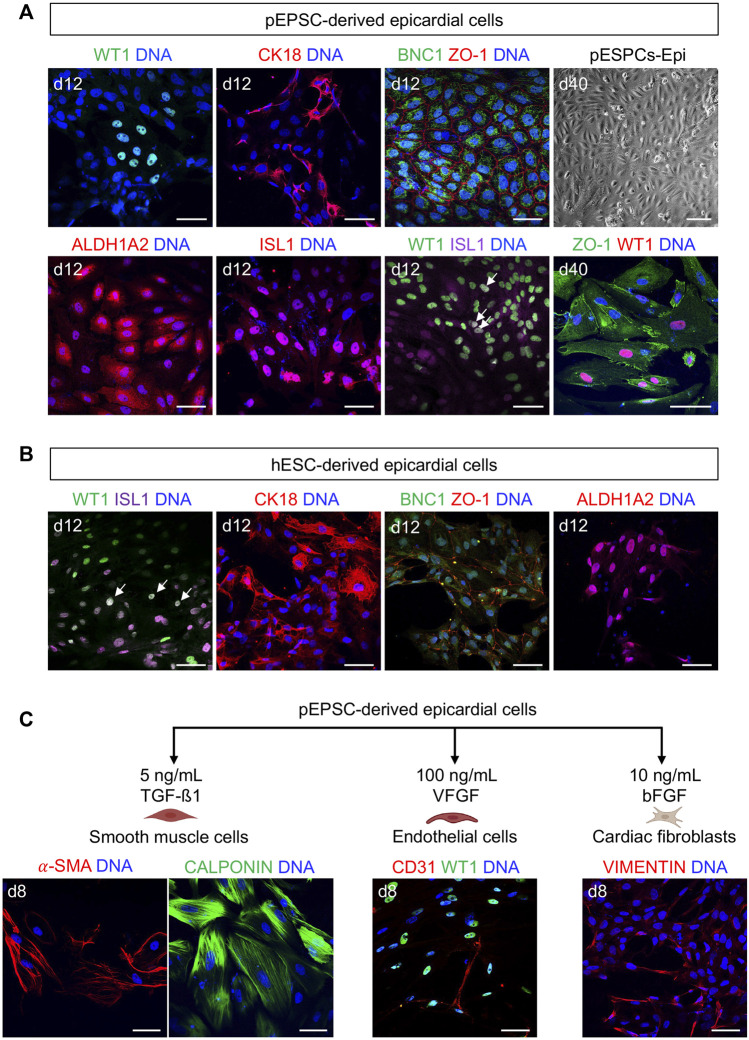
pEPSC-derived epicardial cells express similar epicardial markers to humans. **(A)** Representative bright-field image of pEPSC-derived epicardial cells at day 40 of differentiation displaying typical cobblestone morphology and immunofluorescence images after staining for WT1 (green), CK18 (red), BNC1 (green), ZO-1 (red), ALDH1A2 (red) and ISL1 (red) derived from pEPSCs. Nuclei were labeled with Hoechst 33528 (blue). WT1 (green)/ISL1 (magenta) co-expressing cells are marked with arrows. Scale bars: 50 μm. pEPSCs: porcine expanded potential stem cells. Scale bar: 100 μm, *n* = 6 independent differentiations. **(B)** Representative images of hESC-derived epicardial cells immunostained for ISL1 (magenta), WT1 (green), CK18 (red), BNC1 (green), ZO-1 (red) and ALDH1A2 (red). Nuclei were labeled with Hoechst 33528 (blue). Arrows: cells co-expressing WT1/ISL1. Scale bars: 50 μm, *n* = 1 differentiation. **(C)** Graphical representation of the protocols used to differentiate pEPSC-derived epicardial cells into SMCs, ECs and cardiac FBs. Representative images of SMCs immunostained for CALPONIN (green) and ALPHA SMOOTH MUSCLE ACTIN (α-SMA) (red), ECs stained for CD31 (red) together with epicardial cells stained for WT1 (green) and cardiac FBs stained for fibroblast marker VIMENTIN (red). Scale bars: 50 μm, *n* = 3 independent differentiations.

Having succeeded in applying hPSC-directed cardiac differentiation protocol to pEPSCs, we decided to use a similar approach to generate epicardial cells. We again used the protocol published by Zawada et al. (2023) [based on a modified protocol of Bao et al. (2016)]. This protocol is based on the induction of CPCs from pEPSCs as described above. On day 7, pEPSC-derived CPCs were directed to proepicardial cells by activation of Wnt signaling using GSK-3β inhibitor CHIR for 48 h (Figure 8A). At day 13, pEPSC-derived epicardial cells adopted typical epithelial cobblestone-like morphology similar to human cells (Figure 8B). Molecular analysis revealed progressive induction of well-established epicardial markers *BNC2*, *TBX18*, *ALDH1A2*, *SEMAD3*, *TCF21* and *WT1* during differentiation (Figure 8C), which was consistent with the expression pattern seen in hESC-derived epicardial cells (Bao et al., 2016; Iyer et al., 2016; Guadix et al., 2017). Immunofluorescence analysis showed that pEPSC-derived epicardial cells expressed WT1, cytokeratin 18 (CK18), and BNC1 proteins and formed tight junctions marked by ZO-1 expression along cell borders (Figure 9A). Furthermore, they also expressed aldehyde dehydrogenase enzyme retinaldehyde dehydrogenase 2 (ALDH1A2) (Figure 9A) indicating that these cells could synthesize retinoic acid, which is a sign of more mature functional epicardial cells (Witty et al., 2014). Of note, at day 12, some porcine WT1^+^ cells co-expressed ISL1, resembling the expression pattern of hESC-derived epicardial cells *in vitro* (Figures 9A, B) (Sun et al., 2007). This was in line with our results from the native pig embryos at ED19 (Supplementary Figures S3A, A′) and with recent findings in the early developing epicardium of human embryos and hPSC-derived epicardioids (Meier et al., 2023), confirming a higher degree of similarity between pig and human cardiac development when compared to the mouse. Inhibition of TGF-β signaling by SB431542 enabled expansion of pEPSC-derived epicardial cells, which maintained their epithelial characteristics for more than 40 days (14 passages) (Figure 9A).

The *in vitro* generation of epicardial cells from pEPSCs provides a source of porcine epicardial cells for functional studies aimed at harnessing the regenerative capacity of the epicardium for therapeutic purposes.

### Porcine epicardial cells undergo EMT to differentiate into SMCs, ECs and cardiac FBs

During cardiac development *in vivo*, a subset of epicardial cells undergo epithelial-to-mesenchymal transition (EMT) to become epicardial-derived cells (EPDCs) that migrate into the myocardium and give rise to several differentiated non-myocardial cell types including mural cells (vascular smooth muscle cells and pericytes) and cardiac FBs (Cai et al., 2003; Cai et al., 2008; Bao et al., 2016). However, differentiation into CMs and coronary endothelial cells is still debated (Quijada et al., 2020). Previous studies have shown that both mouse and human *in vitro*-derived epicardial cells possess the same potential (Witty et al., 2014; Iyer et al., 2016; Bao et al., 2017b; Meier et al., 2023).

We adopted the protocol published by Bao et al. (2017a, b) to initiate epicardial EMT *in vitro* from pEPSC-derived epicardial cells and induce SMCs, ECs and FBs differentiation (Figure 9C). SMCs could be differentiated upon TGF-β1 treatment and expressed both CALPONIN and ALPHA SMOOTH MUSCLE ACTININ (α-SMA). Maintaining cultures in the presence of bFGF led to a generation FBs-like cells expressing VIMENTIN, whereas angiogenic growth factor VEGF promoted emergence of ECs expressing CD31 (Figure 9C).

## Discussion

The pig represents one of the large animal models currently used in human disease-related translational research (Romagnuolo et al., 2019; Zhao et al., 2021; Poch et al., 2022). Several studies in the past described pre-gastrulation and gastrulation processes in pigs (Flechon et al., 2004; Oestrup et al., 2009); however, little is known about porcine heart development. Recently, a developmental profile of the cardiovascular system in porcine embryos has been described by Gabriel et al. (2021) and Lauschke et al. (2021), providing the first insights into porcine cardiogenesis. Still, the knowledge of early porcine heart development is very limited and a more detailed, comprehensive, stage-by-stage characterization of molecular determinants is needed. Our study provides a detailed anatomical and molecular analysis of porcine development from the primitive streak stage to the four-chambered beating heart. For the first time, we identified and molecularly characterized porcine proepicardium and epicardium during embryonic development. Our findings highlight previously unappreciated differences between porcine and murine epicardium, which are conserved in human.

In pig, gastrulation starts with the appearance of the primitive streak marked by the expression of TBXT at ED13/ED14 (Hyttel et al., 2011). The earliest cardiac mesoderm progenitors migrate from the primitive streak to form the cardiac crescent (Lescroart et al., 2014; Ivanovitch et al., 2021). We found the first cells marked by cTNT in splanchnic mesoderm in the porcine embryo at ED14, which formed a horseshoe-shaped structure resembling the cardiac crescent. We observed the generation of a linear heart tube at ED15, which is in line with the results published by Lauschke et al. (2021). Porcine ED17 was marked by the presence of PEO, which has not been previously characterized in porcine embryos. By ED19, the porcine heart showed tremendous growth and became four-chambered, corresponding to ED20/ED22 as described by Lauschke et al. (2021).

The expression of pan-cardiac markers ISL1 and NKX2.5 observed in porcine ED14, ED15, ED17 and ED19 overlapped with mouse and human expression pattern (Elliott et al., 2003; Kelly et al., 2014; Zhang et al., 2014; Ren et al., 2021). Importantly, for the first time, we identified and characterized porcine PEO cells expressing WT1 at ED17, showing similar WT1 expression dynamics to humans and mice (Zhou et al., 2008; Risebro et al., 2015). Intriguingly, at ED17 we observed some WT1^+^ and ISL1^+^ cells migrating toward the myocardium and forming the epicardium of the developing heart. This data highlights previously unappreciated differences between pig and mouse epicardial cells, as the latter do not express ISL1 (Sun et al., 2007), and similarity to the human counterpart (Meier et al., 2023). A more detailed analysis will be necessary to fully characterize the origin and differentiation potential of these cells in the pig. It has been shown in mouse that WT1^+^ PEO progenitors are derived from ISL1^+^/NKX2.5^+^ precursors (Zhou et al., 2008) and that epicardial cells give rise to the coronary vasculature and potentially to a small population of myocytes during embryonic and postnatal development (Dorn et al., 2018; Wagner et al., 2021). In human fetal and adult heart WT1^+^ cells were found in epicardium, sub-epicardium, and myocardial layer (Duim et al., 2016).

An improved understanding of porcine cardiac development is essential not only for studying cardiac physiology but also for developing cell-based therapies for preclinical testing. The limited regenerative capacity of the adult heart is insufficient to replace damaged CMs leading to heart failure (Eschenhagen et al., 2017; Tzahor and Poss, 2017). Heart transplantation is usually the only available treatment option for patients with end-stage heart failure but it is limited by the discrepancy between the availability of donors and recipients (Boilson et al., 2010; Tzahor and Poss, 2017). Therefore, there is enormous interest in tissue-engineered porcine biomaterials or cell replacement-based therapies aimed at the repopulation of damaged cardiac tissue in the pig as preclinical model (Cui et al., 2005; Foo et al., 2018; Miia et al., 2021; Maselli et al., 2022). From a clinical perspective, allogeneic and autologous cell therapies may provide a better understanding of the respective immunological responses and the corresponding immunosuppression regimens for future PSC-derived cell therapies in humans (Wu et al., 2012; Stauske et al., 2020). Until now, for testing of allogenic therapies in the porcine heart infarct model, CPCs derived directly from heart biopsy were used (Crisostomo et al., 2015; Prat-Vidal et al., 2021). The disadvantage of these cells is their limited proliferation potential *in vitro* and, thus constant need of replacement by new donor animals. Therefore, exploring the prospects and safety of preclinical cell therapy creates interest in the establishment of cardiac cells directly from pESCs. In the present study, for the first-time pEPSCs could be directed to differentiate into functional cardiac and epicardial lineages, *in vitro*, recapitulating the differentiation potential of hPSCs towards ventricular-like CMs and epicardial cells. Thus, these cells offer a valuable source of pEPSC-derived CPCs, CMs and epicardial cells for future preclinical testing of autologous and allogeneic cardiac cell therapies and would be an authentic reflection of human physiology in preclinical studies. Furthermore, pEPSC-derived CPCs can be functionally validated using *ex utero* porcine embryo culture platform, allowing for faithful recapitulation of porcine cardiac development outside the uterus.

## Data Availability

The original contributions presented in the study are included in the article/Supplementary Material, further inquiries can be directed to the corresponding authors.
