# Supplementary figures and images for: Recapitulating porcine cardiac development in vitro: from expanded potential stem cell to embryo culture models
Source: Front Cell Dev Biol. 2023 May 15;11:1111684. doi: 10.3389/fcell.2023.1111684 (PMC10227949; doi:10.3389/fcell.2023.1111684)

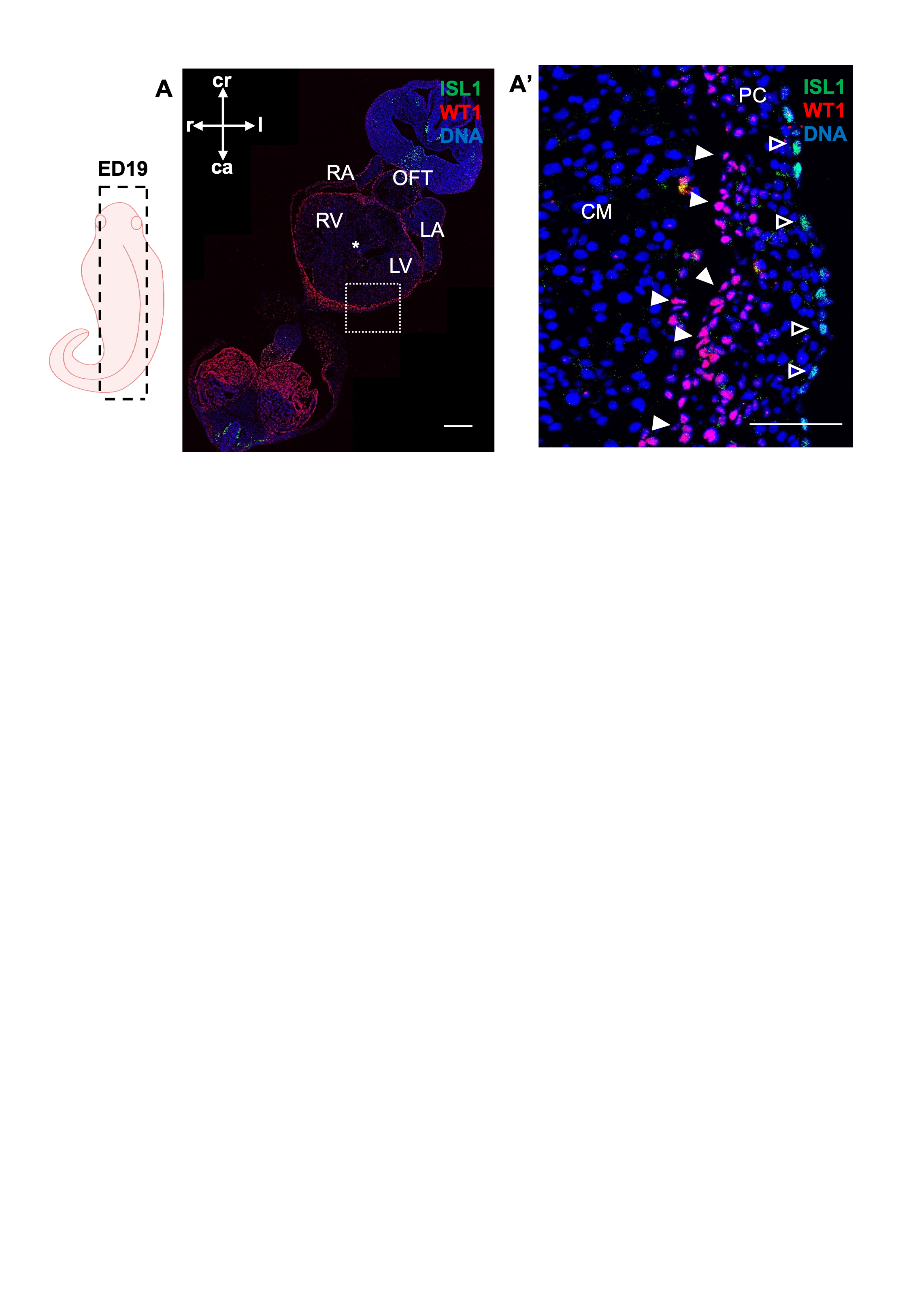

Supplement: Supplementary file 1 [file Image3.TIFF]

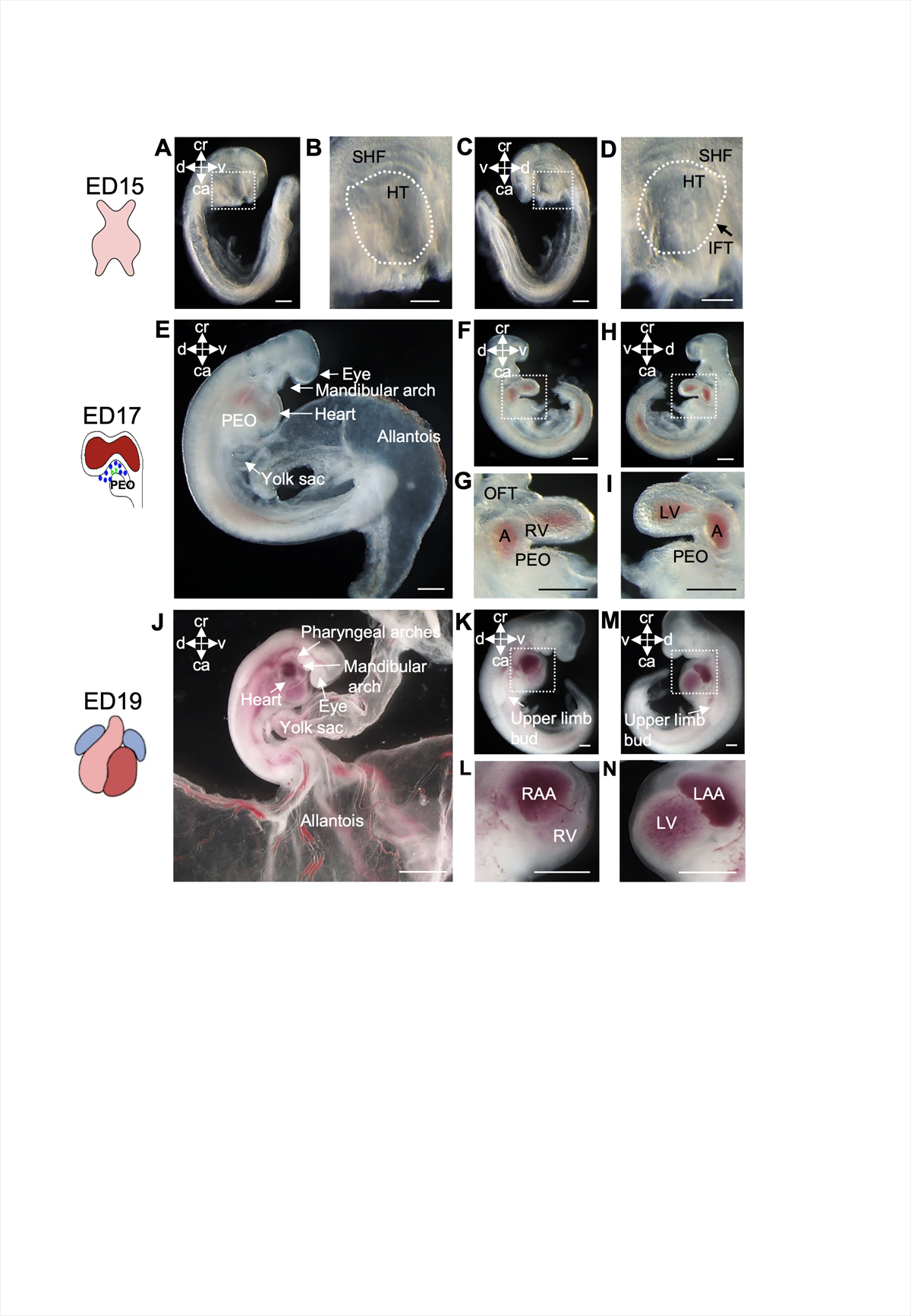

Supplement: Supplementary file 2 [file Image1.tiff]

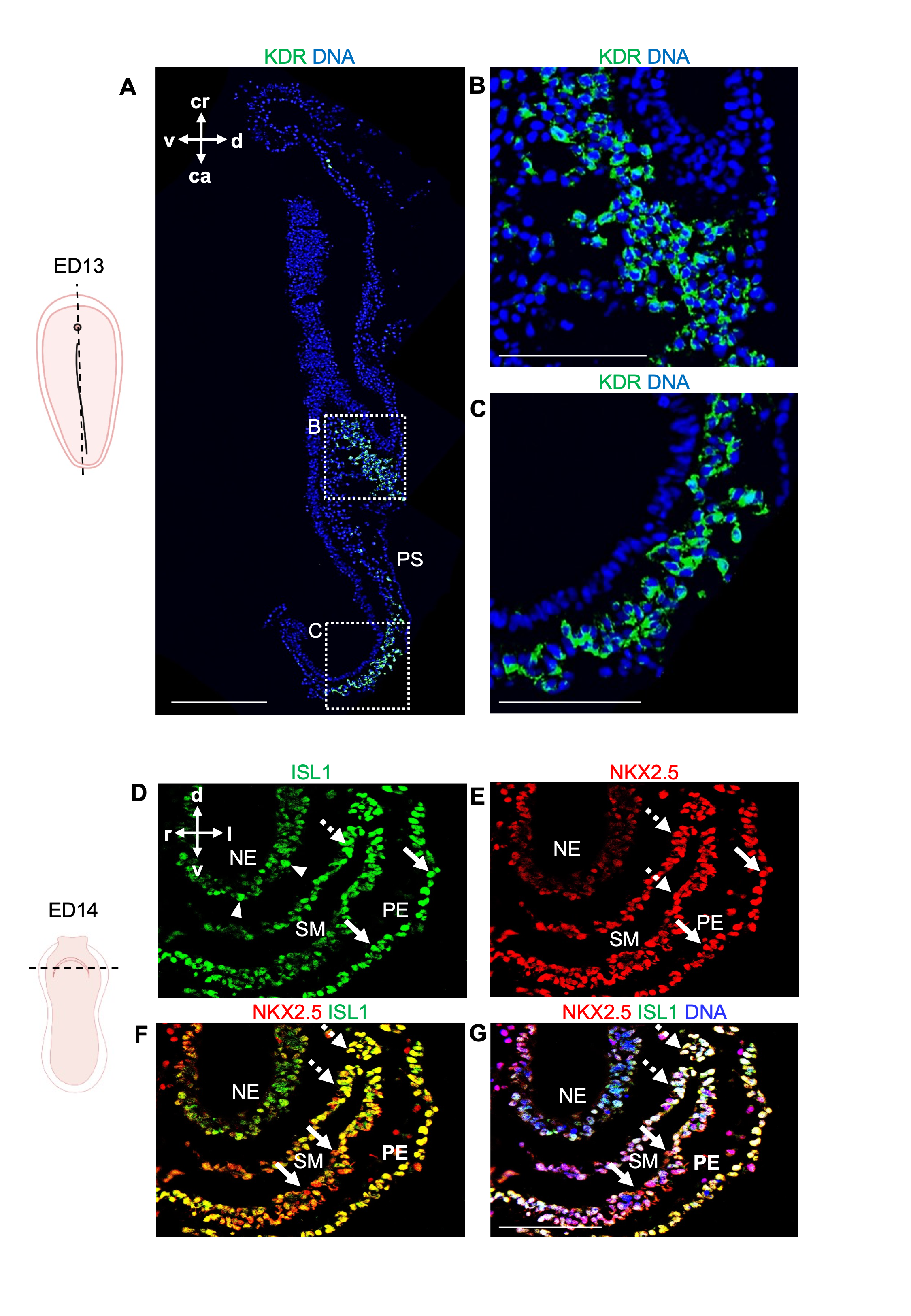

Supplement: Supplementary file 5 [file Image2.TIFF]

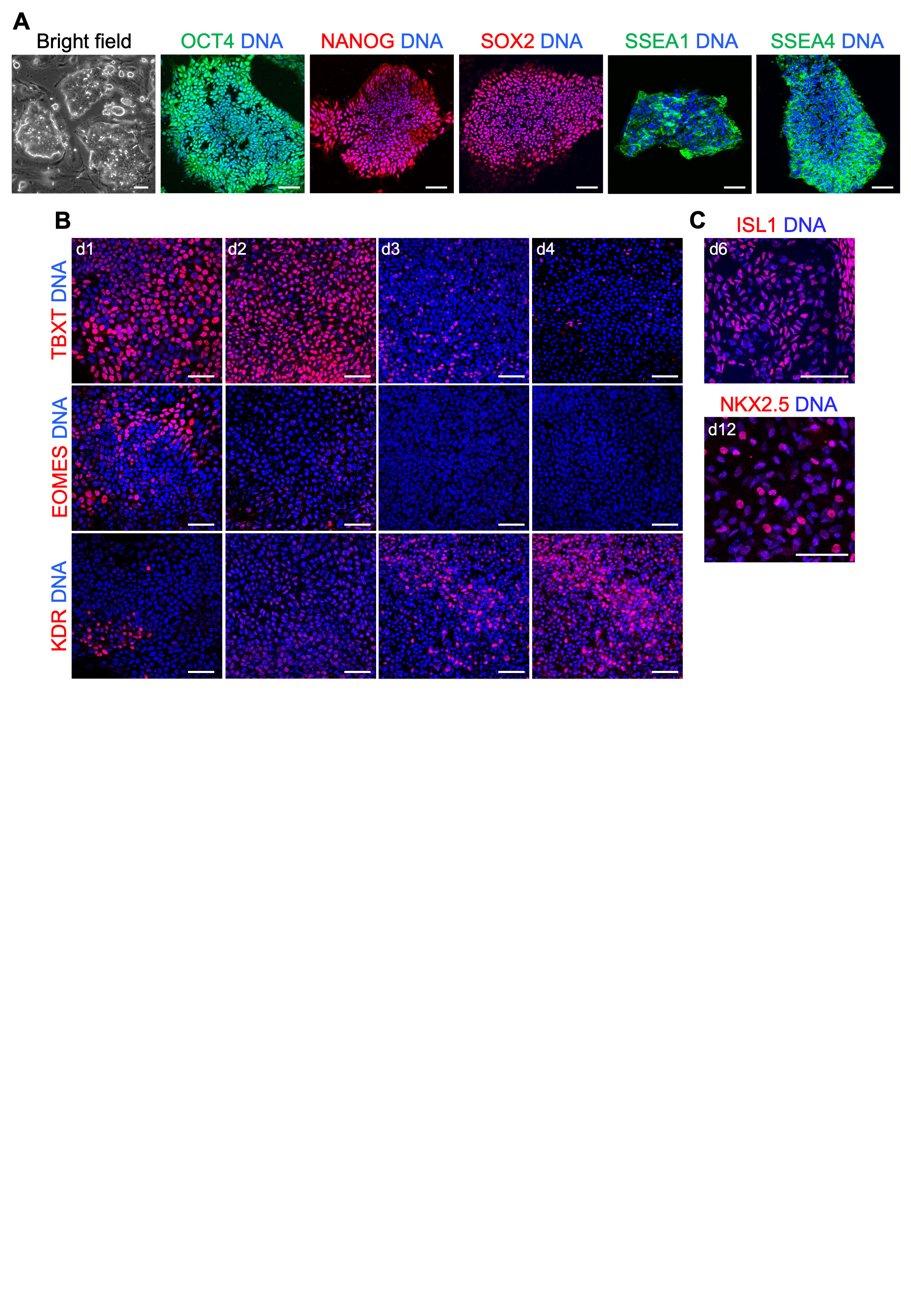

Supplement: Supplementary file 6 [file Image4.TIFF]
