# Supplementary material for: Recapitulating porcine cardiac development in vitro: from expanded potential stem cell to embryo culture models
Source: Front Cell Dev Biol. 2023 May 15;11:1111684. doi: 10.3389/fcell.2023.1111684 (PMC10227949; doi:10.3389/fcell.2023.1111684)
Supplement: Supplementary file 4 [file Table1.pdf]

Supplementary Material

Recapitulating porcine cardiac development *in vitro*: from expanded potential stem cell to embryo culture models

Hilansi Rawat [1,2,3†], Jessica Kornherr[1,2,3†], Dorota Zawada[1,2,3], Sara Bakhshiyeva[1,2,3], Christian Kupatt[1], Karl-Ludwig Laugwitz[1], Andrea Bähr[1], Tatjana Dorn[1,2,3], Alessandra Moretti[1,2,3,4*] and Monika Nowak-Imialek[1,2,3*]

[1]First Department of Medicine, Cardiology, Klinikum rechts der Isar, Technical University of Munich, School of Medicine and Health, Munich, Germany.
[2]German Center for Cardiovascular Research (DZHK), Munich Heart Alliance, Munich, Germany.
[3]Regenerative Medicine in Cardiovascular Diseases, First Department of Medicine, Klinikum rechts der Isar, Technical University of Munich, School of Medicine and Health, Munich, Germany.
[4]Department of Surgery, Yale University School of Medicine; New Haven, USA.

[†]These authors contributed equally.

***Correspondence**: A.M.: amoretti@mytum.de and M.N.I.: monika.nowak-imialek@tum.de

Supplementary Figures

Supplementary Figure S1. Pig embryos at ED15, ED17, and ED19 of development. **(A-D)** Right (A, B) and left (C, D) view of the ED15 pig embryo depicting linear heart tube. The boxed regions in panel A and C are shown at higher magnification in (B) and (D), respectively. The linear heart tube is indicated by the dotted line (B,D). **(E-I)** Lateral view of the ED17 pig embryo. At this stage the looping heart and PEO as well as eyes, mandibular arch, allantois, and yolk sac can be seen (E). The boxed regions in panel F (right view) and H (left view) are shown at higher magnification in (G) and (I), respectively. **(J-N)** Lateral view of the ED19 pig embryo. illustrating the developing heart, eyes, pharyngeal arches, allantois, and yolk sac (E). The boxed regions in panel K (right view) and M (left view) are shown at higher magnification in (L) and (N), respectively. Right and left atrial appendages and both ventricles are clearly

visible (L,N). cr: cranial; ca: caudal; d: dorsal; HT: heart tube; LAA: left atrial appendage; LV: left ventricle; PEO: proepicardiaum organ; RAA: right atrial appendage; RV: right ventricle; LV: left ventricle; v: ventral. Scale bars: 1 mm.

Supplementary Figure S2. Expression of KDR, ISL1 and NKX2.5 in ED13 and ED14 porcine embryos. **(A-C)** Representative image of a sagittal section of ED13 embryo after immunofluorescence analysis of KDR (green). Nuclei were labeled with Hoechst 33528 (blue). Scale bars: 250 µm. The boxed regions in panel A are shown at higher magnification in **(**B**)** and **(**C**)**. Scale bars: 100 µm. **(D-G)** Representative images of transverse section of ED14 embryo after immunofluorescence analysis of ISL1 (green) and NKX2.5 (red). Nuclei were labeled with Hoechst 33528 (blue). ISL1^{+} cells were present in pharyngeal endoderm (arrows), splanchnic mesoderm (dashed arrows), and neuroectoderm (filled arrowheads) (D). NKX2.5^{+} cells were detected in pharyngeal endoderm (arrows) and splanchnic mesoderm (dashed arrows) (E). NKX2.5^{high}/ ISL1^{low} (FHF, arrows) and NKX2.5^{high}/ ISL1^{high} (SHF, dashed arrows) are indicated (F,G). Scale bar: 10 µm. ca: caudal; cr: cranial; d: dorsal; l: left; NE: neuroectoderm; PE: pharyngeal endoderm; PS: primitive streak; r: right; SM: splanchnic mesoderm; v: ventral.

Supplementary Figure S3. Expression of ISL1 and WT1 in ED19 porcine hearts. (A, A') Representative images of sagittal section of ED19 embryo after immunofluorescence analysis of ISL1 (green) and WT1 (red) depicting WT1^{+} cells in the epicardium of the developing heart (filled arrowheads) and few ISL1^{+} cells (empty arrowheads) detected exclusively in the pericardium. No co-expression of ISL1 and WT1 could be detected in these structures. The boxed region in panel A is shown at higher magnification in A'. Nuclei were labeled with Hoechst 33528 (blue). Scale bars: 10 µm. Section corresponds to the position indicated by the lines drawn through the adjacent embryo view. ca: caudal; CM: cardiomyocytes; cr: cranial; l: left; LA: left atrium; LV: left ventricle; OFT: outflow tract; PC: pericardium; r: right; RA: right atrium; RV: right ventricle. Asterix indicates interventricular septum.

Supplementary Figure S4. Differentiation of pEPSCs into early cardiac mesoderm and CPCs. (A) Representative bright-field image of pEPSCs at passage 40 and immunofluorescence images of pEPSCs for the pluripotency factors OCT4 (green), NANOG (red), and SOX2 (red) and cell surface pluripotency markers SSEA1 (green) and SSEA4 (green). Hoechst 33258 (blue) was used to label nuclei. Scale bars: 50 µm. **(B)** Representative images of pEPSC-derived cells stained for early mesoderm marker TBXT (red) and cardiac

mesoderm markers EOMES (red) and KDR (red) during the first four days of differentiation. Nuclei were labeled with Hoechst 33258 (blue). Scale bars: 50 µm. **(C)** Representative images of pEPSC-derived cells stained for ISL1 (red) at day 6 and NKX2.5 (red) at day 12 of differentiation. Hoechst 33258 (blue) was used to label nuclei. Scale bars: 50 µm.

Supplementary Video. Beating cardiomyocytes derived from pEPSCs at day 65 of differentiation.
